# Distinct remission immune architectures under rituximab and azathioprine in AQP4-IgG-positive neuromyelitis optica spectrum disorder

**DOI:** 10.3389/fimmu.2026.1834992

**Published:** 2026-05-08

**Authors:** Wangyong Shin, Bohwan Yoon, Hyo Jae Kim, Dayoung Seo, Inhye Jang, Jihong Ryu, Lynkyung Choi, Jinhee Kim, Hyunjin Kim, Young-Min Lim, Hyung-Seung Jin, Eun-Jae Lee

**Affiliations:** 1 Department of Neurology, Asan Medical Center, University of Ulsan College of Medicine., Seoul, Republic of Korea; 2 Department of Convergence Medicine, Asan Institute for Life Sciences, Asan Medical Center, University of Ulsan College of Medicine, Seoul, Republic of Korea

**Keywords:** azathioprine, IP-10/CXCL10, NMOSD, remission, rituximab, TIGIT

## Abstract

**Introduction:**

Maintenance immunotherapy is effective in AQP4-IgG-positive neuromyelitis optica spectrum disorder (NMOSD), but how mechanistically distinct maintenance therapies organize the remission immune landscape and its relationship to neurological disability remains poorly defined.

**Methods:**

We performed integrated multiparameter flow cytometry and plasma cytokine profiling in serial remission samples (up to three per patient) from 28 patients receiving rituximab (RTX, n = 14) or azathioprine (AZA, n = 14). Between-treatment comparisons, within-treatment coupling analyses, and disability-associated immune analyses were conducted using age-adjusted patient-clustered regression models with prespecified false discovery rate control.

**Results:**

RTX showed clear target engagement, characterized by profound B-cell depletion, gradual post-infusion reconstitution, and sustained reduction of natural killer T (NKT)-like cells. Beyond lineage depletion, RTX was associated with regulatory remodeling, including a memory-skewed regulatory T (Treg) phenotype and a lower effector-to-regulatory balance. In B-detectable RTX samples, the reconstituting B-cell compartment was transitional/naive-skewed with marked suppression of memory B cells. Although remission-phase cytokines were broadly low, interferon gamma-induced protein 10/CXC motif chemokine ligand 10 (IP-10/CXCL10) remained selectively elevated under RTX. Importantly, remission immune architecture differed by therapy: AZA showed a T cell immunoreceptor with Ig and ITIM domains (TIGIT)-linked regulatory disability axis, whereas RTX showed disability coupling to soluble inflammatory mediators, particularly interleukin-6 (IL-6) and IP-10.

**Conclusion:**

Mechanistically distinct maintenance therapies impose divergent remission immune architectures in NMOSD. These findings support a treatment-aware framework for biomarker interpretation and suggest that remission monitoring should consider therapy-specific immune networks rather than isolated immune markers.

## Introduction

Neuromyelitis optica spectrum disorder (NMOSD) is a relapsing autoimmune inflammatory disease of the central nervous system that predominantly affects the optic nerves and spinal cord, often leading to irreversible neurological disability (1–3). The identification of pathogenic aquaporin-4 immunoglobulin G (AQP4-IgG) established NMOSD as an autoantibody-mediated astrocytopathy distinct from multiple sclerosis and provided a mechanistic rationale for targeted immunotherapy (1, 4–7). Recent randomized clinical trials have demonstrated substantial relapse reduction with biologics targeting complement and the interleukin-6 (IL-6) receptor, illuminating key immune pathways in NMOSD pathogenesis (1, 8–11).

Despite these advances, rituximab (RTX) and azathioprine (AZA) remain the most widely used maintenance therapies in many regions, driven by established clinical experience, cost, and accessibility. These agents represent mechanistically distinct strategies: RTX selectively depletes CD20^+^ B cells while sparing antibody-secreting plasmablasts, whereas AZA exerts broad antiproliferative effects through thiopurine-mediated inhibition of lymphocyte proliferation. Previous studies have consistently reported superior relapse prevention with RTX compared with AZA, yet the immunological basis for this difference during remission, when long-term stability is maintained, remains incompletely understood (12–16).

The benefits of B-cell depletion likely extend beyond a reduction of circulating AQP4-IgG precursors. Multiple immune pathways implicated in NMOSD pathogenesis converge on B-cell biology, including IL-6–linked plasmablast survival, T helper 17 (Th17) polarization, and T follicular helper (Tfh)–mediated B-cell activation (17–21). RTX-mediated depletion of CD20^+^ B-cell hubs, followed by time-dependent reconstitution, may therefore remodel broader immune circuits rather than simply reducing autoantibody titers (1, 22–24). Innate and innate-like lymphocyte populations, including natural killer (NK) and NKT-like cells, may further shape inflammatory tone and immune regulation, but how these components integrate within distinct maintenance regimens remains poorly defined (25–27).

Prior studies addressing these questions have largely focused on relapse-phase inflammation, individual immune compartments in isolation, or limited biomarker panels, leaving substantial gaps in understanding how coordinated cellular and soluble mediator networks are organized during remission (28–31). Direct comparisons of how mechanistically divergent maintenance strategies such as RTX and AZA structure the remission immune landscape and how that landscape relates to disability remain scarce.

Here, we addressed this gap by integrating multiparameter flow-cytometric immunophenotyping with plasma cytokine profiling in serial remission samples from AQP4-IgG–positive NMOSD patients receiving RTX or AZA. Using age-adjusted, patient-clustered models, we tested whether mechanistically distinct maintenance therapies impose different remission immune architectures, whether these therapies generate divergent cytokine–cell coupling structures, and whether disability maps onto distinct immune axes depending on the treatment context.

## Materials and methods

### Participants

Since June 2018, patients with NMOSD visiting the Department of Neurology at Asan Medical Center (Seoul, Korea) have been prospectively enrolled after providing informed consent (32). The diagnosis of NMOSD was established according to consensus criteria (2). For the present study, only patients with AQP4-IgG positivity confirmed by a cell-based assay were included. Blood samples were generally collected at 6–12-month intervals during remission. For patients receiving RTX, samples were collected at variable time points after infusion according to clinic visit schedules. In cases of clinical relapse, additional samples were obtained at the time of relapse and during the subsequent remission period; only remission-phase samples were used for the present analyses. For RTX treatment, induction consisted of two 1,000 mg doses administered 2 weeks apart, followed by maintenance dosing with a single 1,000 mg infusion when CD19^+^ B cells exceeded 1% or CD19^+^CD27^+^ memory B cells exceeded 0.05% of peripheral blood mononuclear cells (PBMCs) (33).

Patients were retrospectively selected from an ongoing prospective NMOSD cohort (2018–2023). Among the 134 registered patients, those who consented to PBMC collection and had at least two remission-phase samples were considered eligible. Patients with fewer than two remission-phase samples or without consent for PBMC collection were excluded.

RTX and AZA were selected as comparator groups because they represented the majority (75%) of maintenance therapies in our cohort during the study period, whereas other treatments such as mycophenolate mofetil (MMF) were not included due to limited availability of longitudinal PBMC samples. For RTX users, only those on maintenance dosing were included to ensure consistent therapeutic exposure.

**Figure 1:**
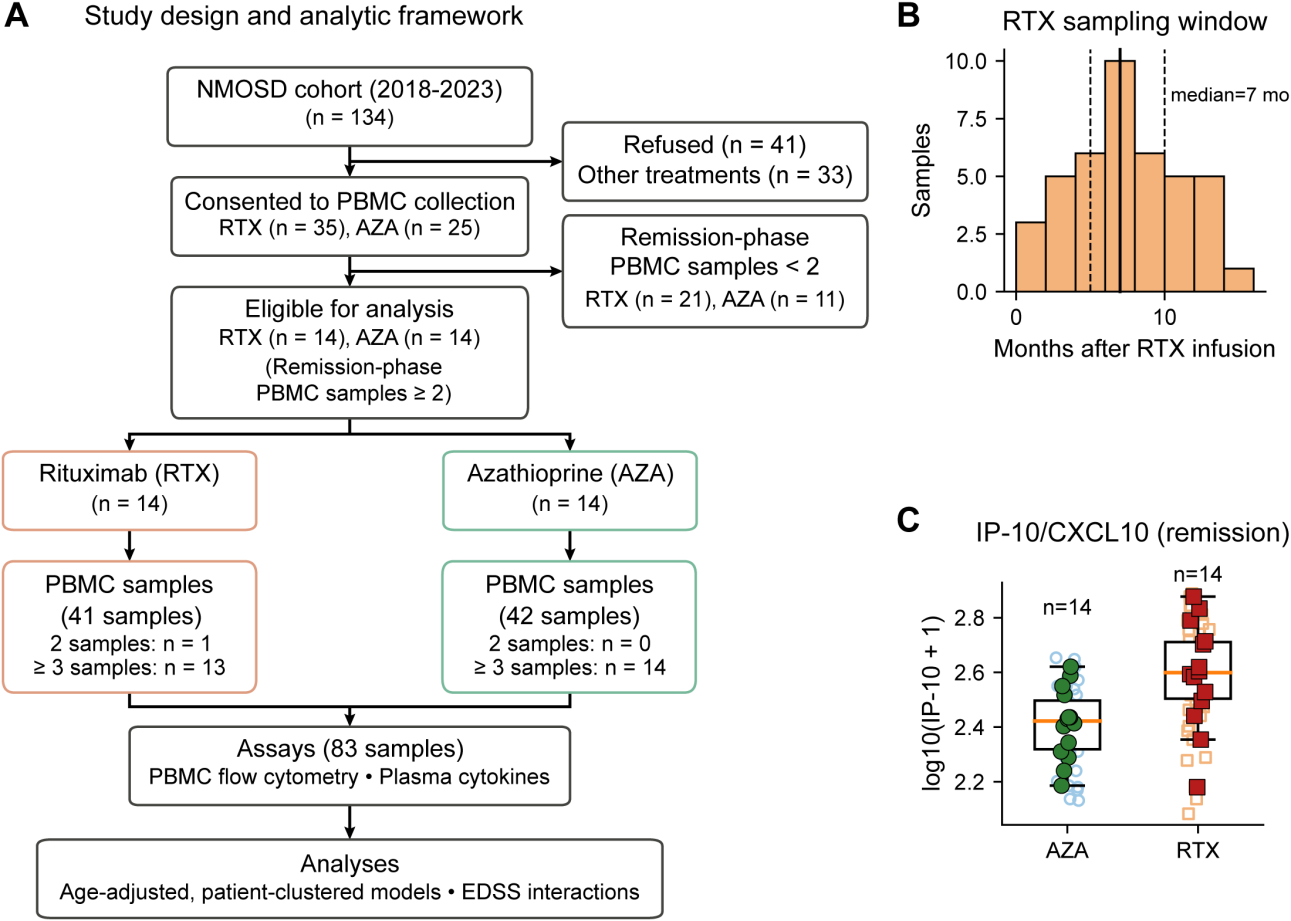
Study design and analysis overview. **(A)** Cohort and sampling framework for remission-phase profiling of NMOSD patients receiving RTX or AZA, including serial remission sampling. **(B)** Distribution of sampling times (months) after the most recent RTX infusion among RTX remission samples. **(C)** Remission-phase IP-10/CXCL10 distributions according to maintenance therapy.Where applicable, light symbols represent individual remission samples and filled symbols represent patient-level medians.

A total of 28 patients (14 RTX and 14 AZA) met inclusion criteria and contributed serial remission-phase samples. The number of samples per patient varied, with most patients contributing three or more samples. Details of cohort selection and sampling structure are summarized in **Figure 1A**. The present study represents a retrospective nested analysis of patients and serial remission-phase samples derived from an ongoing prospective NMOSD cohort/biobank.

### Blood sample processing

Whole blood was diluted with phosphate-buffered saline (PBS) and layered onto Ficoll-Paque Premium (Cytiva) for density gradient separation. Plasma was collected and stored at -80 °C. PBMCs were washed and cryopreserved in CELLBANKER1 medium (ZENOAQ). Cells were cooled at a controlled rate to -80 °C using CoolCell containers (CORNING) and subsequently transferred to vapor-phase liquid nitrogen (-192 °C) for long-term storage.

### Flow cytometry

Cryopreserved PBMCs were thawed rapidly at 37 °C, and viability was assessed using Zombie NIR Fixable Viability Dye (BioLegend). Fc receptors were blocked with Human TruStain FcX (BioLegend) before antibody incubation. For surface staining, cells were incubated at 4 °C for 20 minutes in FACS buffer (PBS with 1% BSA and 0.1% sodium azide) with pre-optimized antibody cocktails. For intracellular staining, surface-labeled cells were fixed and permeabilized using the FoxP3/Transcription-Factor Staining Buffer Set (Invitrogen), followed by staining in Perm/Wash buffer (BD Biosciences).

The following fluorochrome-conjugated antibodies were used: anti-human CD3 (clone SK7, BV510, BioLegend), anti-human CD4 (clone SK3, BV786, BD Biosciences), anti-human CD14 (clone 63D3, APC, BioLegend), anti-human CD16 (clone 3G8, PE, BioLegend), anti-human CD19 (clone HIB19, PE-Dazzle 594, BioLegend), anti-human CD24 (clone ML5, PerCP-Cy5.5, BD Biosciences), anti-human CD25 (clone M-A251, PE-Cy7, BioLegend), anti-human CD27 (clone O323, VioletFluor 450, Tonbo Biosciences), anti-human CD38 (clone HB-7, FITC, BioLegend), anti-human CD45RA (clone HI100, APC, Tonbo Biosciences), anti-human CD45RO (clone UCHL1, PE-Dazzle 594, BioLegend), anti-human CD56 (clone 5.1H11, PerCP-Cy5.5, BioLegend), anti-human CD127 (clone A019D5, BV605, BioLegend), anti-human CD183 (CXCR3; clone G025H7, APC, BioLegend), anti-human CD185 (CXCR5; clone RF8B2, Alexa Fluor 488, BD Biosciences), anti-human CD194 (CCR4; clone 205410, FITC, R&D Systems), anti-human CD196 (CCR6; clone G034E3, PE, BioLegend), anti-human CD197 (CCR7; clone G043H7, PerCP-Cy5.5, BioLegend), anti-human CD226 (clone 11A8, PE-Cy7, BioLegend), anti-human CD226 (clone 11A8, BV421, BioLegend), anti-human CCR10 (clone 314305, APC, R&D Systems), anti-human T cell immunoreceptor with Ig and ITIM domains (TIGIT; clone A15153G, BV605, BioLegend), and anti-human FOXP3 (clone 259D, PE, BioLegend). Data were acquired on a CytoFLEX flow cytometer (Beckman Coulter, Brea, CA, USA) and analyzed using FlowJo software (version 10.5.3, Tree Star). A representative gating strategy is provided in **Supplementary Figure 1**. B cells were identified using CD19 as the primary gating marker rather than CD20. Because rituximab targets CD20, its use as a detection marker following RTX treatment is limited by potential epitope masking, antigen internalization, and depletion of CD20-expressing cells, which may lead to underestimation of residual B cells (34). In contrast, CD19 is not directly targeted by RTX and remains reliably detectable on residual and reconstituting B cells (35).

Reporting units/parent denominators were prespecified: major leukocyte populations (CD19^+^ B, CD3^+^ T, CD25^+^ Treg, NKT-like, NK, monocytes) are reported as % of live CD45^+^ cells; naive/memory CD4 and CD8 as % within CD4 or CD8 T cells; naive/memory Treg as % within CD4^+^ Treg; helper subsets as % within conventional CD4; B subsets (naive, memory, transitional, plasmablast) as % within CD19^+^ B; and TIGIT/CD226 quadrants as % within memory Treg. Because RTX induces profound B-cell depletion, B-subset phenotyping was available only in B-detectable (subset-evaluable) samples; subset analyses were therefore performed on the evaluable subset, and evaluability was additionally modeled as described below.

### Cytokine measurements

Plasma samples were stored at -80 °C and thawed only once immediately prior to analysis. IL-6 and IL-10 were quantified in duplicate using a SIMOA HD-1 Analyzer (Quanterix, MA, USA) by an investigator blinded to clinical data. Ten additional cytokines (granulocyte macrophage colony-stimulating factor (GM-CSF), interferon gamma (IFN-γ), IL-1β, IL-2, IL-4, IL-8, IL-13, IL-17A, interferon gamma-induced protein 10/CXC motif chemokine ligand 10 (IP-10/CXCL10), tumor necrosis factor alpha (TNF-α)) were measured using the Milliplex system (Merck) at Bioinfra (Seoul, South Korea), also by blinded investigators.

### Statistical analysis

Continuous variables were summarized as medians with interquartile ranges and compared using the Mann-Whitney U test. Categorical variables were compared using the Chi-square test.

For immune-cell and cytokine outcomes measured in serial remission samples, between-treatment comparisons were performed using age-adjusted marginal regression models with patient-clustered robust standard errors to account for repeated sampling (patient as the clustering unit). For flow-cytometry outcomes analyzed on the percentage scale, effects are reported as differences in percentage points (ΔRTX-AZA). Cytokines were analyzed on the log10(x + 1) scale and reported as Δlog10 with corresponding fold-change (10^Δ). Within the RTX cohort, time-after-infusion associations were evaluated using RTX-restricted, age-adjusted patient-clustered models with months after the most recent infusion as a continuous predictor.

**Figure 2:**
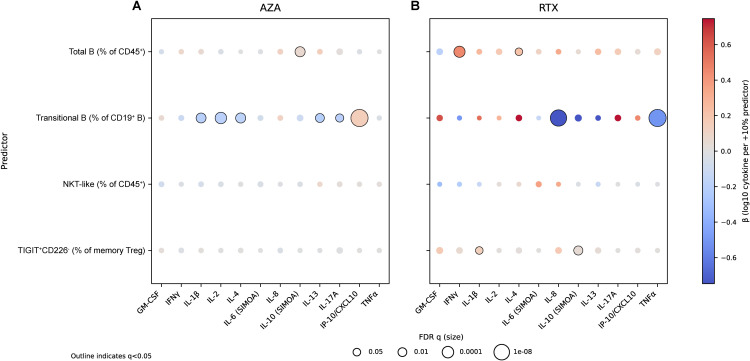
Therapy-specific cytokine–cell coupling networks during remission. Bubble matrices summarize within-treatment, age-adjusted, patient-clustered regression models of log10(cytokine + 1) as a function of key immune subsets. Rows indicate cellular predictors (Total B [% of CD45^+^], Transitional B [% of CD19^+^ B], NKT-like [% of CD45^+^], and TIGIT^+^CD226^-^ memory Treg [% of memory Treg]), and columns indicate cytokines (GM-CSF, IFNγ, IL-1β, IL-2, IL-4, IL-6 (SIMOA), IL-8, IL-10 (SIMOA), IL-13, IL-17A, IP-10/CXCL10, and TNFα). **(A)** AZA. **(B)** RTX. Dot color indicates the signed regression coefficient β (per +10 percentage-point increase in the predictor). Dot size increases as the FDR-adjusted q value decreases, such that larger dots indicate stronger evidence; outlined dots indicate q < 0.05. q values were derived using Benjamini-Hochberg correction within each treatment across all 48 prespecified edges. Analyses involving Transitional B in RTX were restricted to the B-detectable subset.

**Figure 3:**
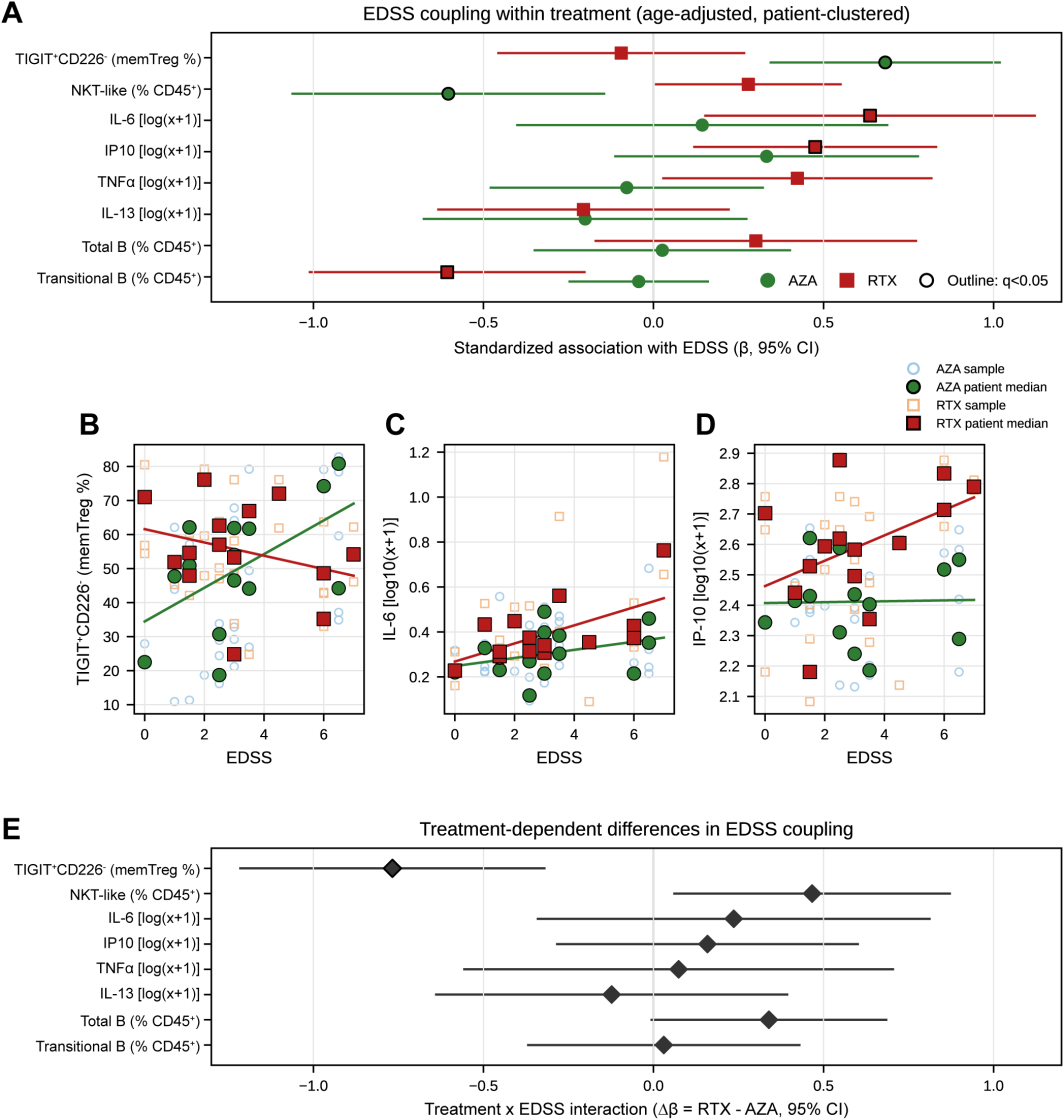
Disability-associated immune axes differ by therapy and are supported by treatment × EDSS interaction. EDSS coupling was assessed using age-adjusted, patient-clustered models. Standardized coefficients were obtained by z-scoring EDSS and each immune marker within each treatment group; β therefore represents the SD change in the immune marker per 1-SD increase in EDSS. Cytokines were analyzed on the log10(x + 1) scale prior to standardization. **(A)** Forest plot of EDSS associations within each treatment (AZA, green circles; RTX, red squares); outlined markers indicate q < 0.05 after Benjamini-Hochberg FDR correction within each treatment across the prespecified EDSS feature set (m = 8). **(B–D)** Representative scatter plots of EDSS versus key markers with fitted within-treatment regression lines shown for visualization: **(B)** TIGIT^+^CD226^-^ (% of memory Treg), **(C)** IL-6, and **(D)** IP-10/CXCL10. Open symbols denote individual samples and filled symbols denote patient-level medians. Statistical inference was based on age-adjusted, patient-clustered models. **(E)** Treatment × EDSS interaction estimates (Δβ = βRTX - βAZA) with 95% confidence intervals for the same feature set; interaction q values reflect FDR correction across the same 8 tests. Analyses involving Transitional B in RTX were restricted to the B-detectable subset. Full model outputs are provided in **Supplementary Table 4A and S4B**, with additional robustness models in **Supplementary Table 5**.

Therapy-specific cytokine–cell coupling (**Figure 2**; **Supplementary Tables 3A**, **S3B**) was assessed within each treatment group using age-adjusted, patient-clustered models of log10(cytokine + 1) as a function of prespecified cellular predictors (total B [%CD45^+^], transitional B [%CD19^+^], NKT-like [%CD45^+^], and TIGIT^+^CD226^-^ memory Treg [%memory Treg]); cellular predictors were scaled per 10 percentage-point increase for interpretability. Disability coupling (**Figure 3**; **Supplementary Table 4A**, **S4B**) was assessed within each treatment group using age-adjusted, patient-clustered models. Standardized coefficients were obtained by z-scoring Expanded Disability Status Scale (EDSS) and each immune marker within each treatment group; β therefore represents the SD change in the immune marker per 1-SD increase in EDSS. Treatment × EDSS interactions were tested in pooled models including main effects for treatment and EDSS and an interaction term; interaction effects are reported as differences in standardized slopes (ΔβRTX-AZA).

To address conditioning on B-subset evaluability under RTX, B-detectability (subset-evaluable vs not) was modeled in RTX-only analyses using age-adjusted patient-clustered binomial regression as a function of months after infusion (**Supplementary Figure 2**; **Supplementary Table 5**). Additional prespecified robustness models (**Supplementary Table 5**) tested whether the RTX-AZA difference in IP-10 remained after adjusting for major cellular shifts (total B and NKT-like), and whether RTX EDSS-cytokine coupling (IL-6, IP-10) remained after adjusting for total B burden or months after infusion.

Two-sided p values are reported throughout. For prespecified multi-outcome analysis families, statistical significance was defined as Benjamini-Hochberg false discovery rate (FDR) q < 0.05. FDR was controlled within the following families: (i) between-treatment flow-cytometry outcomes (**Supplementary Table 1A**; 24 outcomes), (ii) between-treatment cytokines (**Supplementary Table 1B**; 12 cytokines), (iii) within-RTX months-after-infusion analyses across the same flow panel (**Supplementary Table 1C**; 24 outcomes), (iv) patient-level median sensitivity analyses (**Supplementary Table 2A**, **S2B**; flow and cytokines analyzed separately), (v) within-treatment cytokine–cell coupling edges (**Supplementary Table 3A**, **S3B**; 48 edges within each treatment), (vi) within-treatment EDSS feature sets (**Supplementary Table 4A**; 8 features per treatment), and (vii) treatment × EDSS interaction tests across the same 8-feature set (**Supplementary Table 4B**; 8 tests). Robustness models in **Supplementary Table 5** are reported with nominal p values without multiplicity correction. Analyses were performed using IBM SPSS Statistics 28.0.0.0(190), R version 4.4.1 and RStudio version 2024.09.0 + 375. No imputation was performed, and model-specific sample sizes reflect available data for each outcome. Figures were created using Adobe Illustrator 30.3 (Adobe Inc., USA).

## Results

### Patient characteristics and remission-phase cytokine context

**Figure 4:**
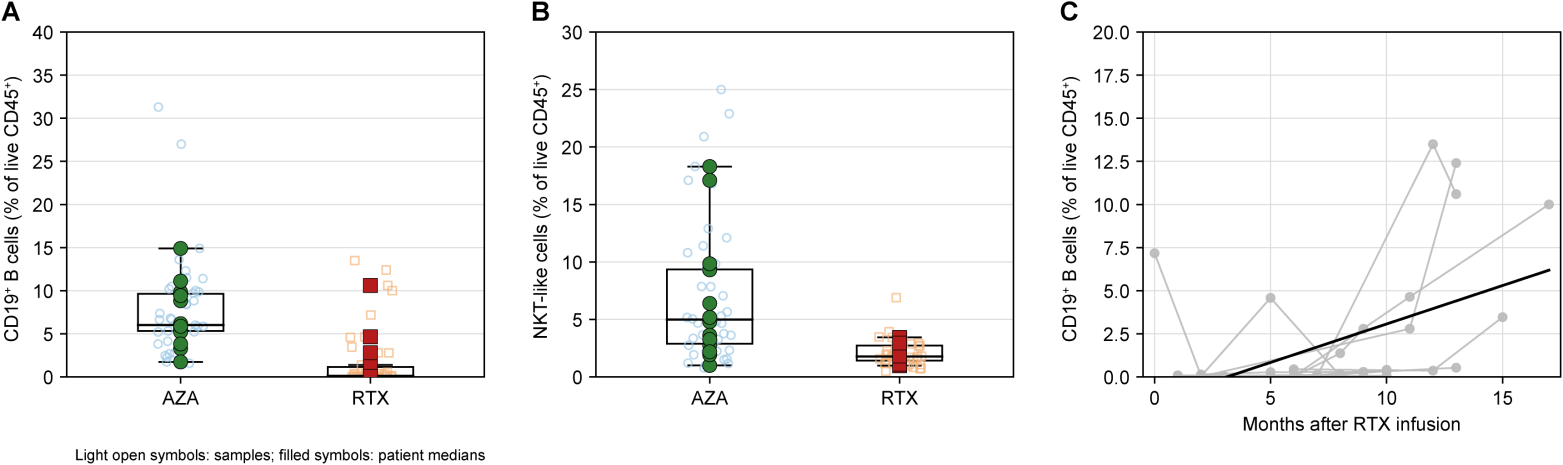
RTX target engagement during remission: B-cell depletion, reduced NKT-like cells, and time-dependent B-cell reconstitution. **(A)** Frequency of CD19^+^ B cells among live CD45^+^ leukocytes in AZA- and RTX-treated patients during remission. **(B)** Frequency of NKT-like cells (CD3^+^CD56^+^) among live CD45^+^ leukocytes in AZA versus RTX during remission. **(C)** RTX kinetics of CD19^+^ B-cell frequency plotted against months after the most recent RTX infusion; gray lines indicate within-patient trajectories across serial remission samples, and the solid black line indicates the fitted trend corresponding to the age-adjusted, patient-clustered slope. For **(A, B)**, light open symbols represent individual remission samples and filled symbols represent patient-level medians; boxplots show median and interquartile range with whiskers at 1.5 × IQR.

Study design, sampling framework, and the analysis overview are summarized in **Figure 1A**. A total of 28 patients with NMOSD in clinical remission were analyzed (RTX, n = 14; AZA, n = 14), with up to three serial remission samples per patient, yielding 83 remission-phase samples overall. Baseline clinical characteristics are summarized in **Table 1**. Median EDSS was comparable between groups, and last attack locations were similarly distributed, with optic neuritis and transverse myelitis predominating. The AZA group had a higher age at onset and older age at sampling, and subsequent regression-based comparisons therefore incorporated age as a covariate (**Supplementary Tables 1A–C**). In the RTX cohort, the time from the most recent infusion to blood draw spanned early and late post-infusion windows (**Figure 1B**); the median time from infusion to blood draw was 7 months (sample-level IQR 5 to 10 months; patient-level median 7 months [IQR 6 to 10.3]). This dispersion enabled evaluation of time-dependent target engagement patterns within RTX (**Figure 4C**; **Supplementary Table 1C**). Plasma cytokine concentrations were generally low, consistent with remission (**Table 1**). Descriptively, IL-6 tended to be higher and IL-10 tended to be lower in RTX compared with AZA (**Table 1**). However, when accounting for age and repeated sampling in patient-clustered models, IP-10/CXCL10 was the only cytokine that remained significantly higher under RTX (Δlog10 = 0.171, 95% CI 0.059 to 0.283; ~1.48-fold RTX/AZA; FDR q = 0.033; **Figure 1C**; **Supplementary Table 1B**). Other cytokines showed directional differences but did not remain significant after multiplicity correction in the adjusted framework (**Supplementary Table 1B**).

**Table 1:** Clinical characteristics and remission-phase cytokine context of patients with NMOSD treated with RTX and AZA.

Clinical characteristics	Rituximab	Azathioprine	p
	(n = 14)	(n = 14)	
Age, median [IQR]	44.5 [38.8-53.0]	58.0 [46.3-66.8]	0.140
Age at onset, median [IQR]	33.0 [26.5-37.5]	46.5 [40.5-55.0]	0.028
Female, n (%)	11 (78.6%)	12 (85.7%)	0.622
Disease duration, median [IQR]	10.0 [5.8-19.0]	6.0 [2.0-11.0]	0.166
Annualized relapse rate, median [IQR]	0.55 [0.32-0.94]	0.33 [0.17-0.71]	0.077
Last attack location, n (%)			0.787
ON	4 (28.6)	4 (28.6)	
TM	8 (57.1)	9 (64.3)	
Brain	1 (7.1)	1 (7.1)	
ON + TM	1 (7.1)	0	
EDSS, median [IQR]	3.0 [1.0-5.0]	3.0 [1.5-4.0]	0.921
Cytokine analysis			
IL-6*, pg/mL, median [IQR]	1.28 [1.02-1.70]	1.05 [0.68-1.54]	0.023
IL-10*, pg/mL, median [IQR]	5.59 [4.24-7.68]	7.49 [5.88-10.47]	0.001
GM-CSF, pg/mL, median [IQR]	0.02 [0.00-4.43]	0.03 [0.00-2.15]	0.998
IFNγ, pg/mL, median [IQR]	2.74 [1.57-8.12]	3.41 [2.11-5.57]	0.418
IL-1β, pg/mL, median [IQR]	0.88 [0.61-2.15]	1.32 [0.67-4.21]	0.113
IL-2, pg/mL, median [IQR]	0.37 [0.27-0.60]	0.28 [0.14-1.05]	0.226
IL-4, pg/mL, median [IQR]	0.68 [0.57-1.02]	0.67 [0.44-1.05]	0.801
IL-8, pg/mL, median [IQR]	6.55 [3.87-8.22]	6.15 [4.52-8.75]	0.837
IL-13, pg/mL, median [IQR]	6.11 [3.40-19.47]	8.19 [3.52-25.96]	0.481
IL-17A, pg/mL, median [IQR]	0.70 [0.40-1.25]	0.48 [0.28-0.85]	0.081
IP-10, pg/mL, median [IQR]	411.49 [282.28-560.05]	260.05 [215.46-332.38]	<0.001
TNFα, pg/mL, median [IQR]	24.82 [20.91-29.43]	24.14 [18.00-33.55]	0.524

RTX, rituximab; AZA, azathioprine; EDSS, Expanded disability status scale; GM-CSF, granulocyte-macrophage colony-stimulating factor; IFNγ, interferon gamma; IL, interleukin; IP-10 interferon gamma-induced protein 10; NMOSD, neuromyelitis optica spectrum disorder; ON, optic neuritis; SIMOA, single molecule array; TM, transverse myelitis; TNFα, tumor necrosis factor alpha.*Measured by Single Molecule Array (SIMOA).

### RTX target engagement: profound B-cell depletion, time-dependent reconstitution, and reduced NKT-like cells

Multiparameter flow-cytometry profiling (**Supplementary Figure 1**) demonstrated pronounced regimen-dependent remodeling of the CD45^+^ immune landscape. The most robust between-group difference was CD19^+^ B-cell depletion under RTX (**Figure 4A**). At the patient level, CD19^+^ B cells were markedly lower in RTX compared with AZA. In age-adjusted patient-clustered models, RTX remained strongly associated with reduced CD19^+^ B frequency (ΔRTX-AZA = -6.98 percentage points, 95% CI -9.63 to -4.33; p = 2.42 × 10^-7^; FDR q = 1.94 × 10^-6^; **Supplementary Table 1A**). Within RTX, CD19^+^ B frequency increased with months after infusion in adjusted models (**Figure 4C**; **Supplementary Table 1C**), consistent with gradual reconstitution between maintenance doses. In age-adjusted patient-clustered RTX-only models, the CD19^+^ B fraction increased by +0.44 percentage points per month after infusion (95% CI 0.08 to 0.80; p = 0.0165; **Supplementary Table 1C**). A second robust cellular signature of RTX was a lower frequency of NKT-like cells (CD3^+^CD56^+^) during remission (**Figure 4B**). In age-adjusted patient-clustered models, NKT-like frequency remained significantly lower with RTX (ΔRTX-AZA = -4.92 percentage points, 95% CI -7.61 to -2.23; p = 3.44 × 10^-4^; FDR q = 0.00206; **Supplementary Table 1A**). Unlike B cells, NKT-like frequencies showed no significant association with months after RTX infusion (p = 0.195; **Supplementary Table 1C**), consistent with a sustained reduction rather than a short-lived post-infusion effect. Other broad leukocyte compartments were comparatively stable between regimens after age adjustment (**Supplementary Table 1A**), indicating that the most prominent remission-phase differences were concentrated in the B-cell axis and the NKT-like compartment.

### Reconstituting B cells under RTX are transitional/naive-skewed, with marked suppression of memory B cells

**Figure 5:**
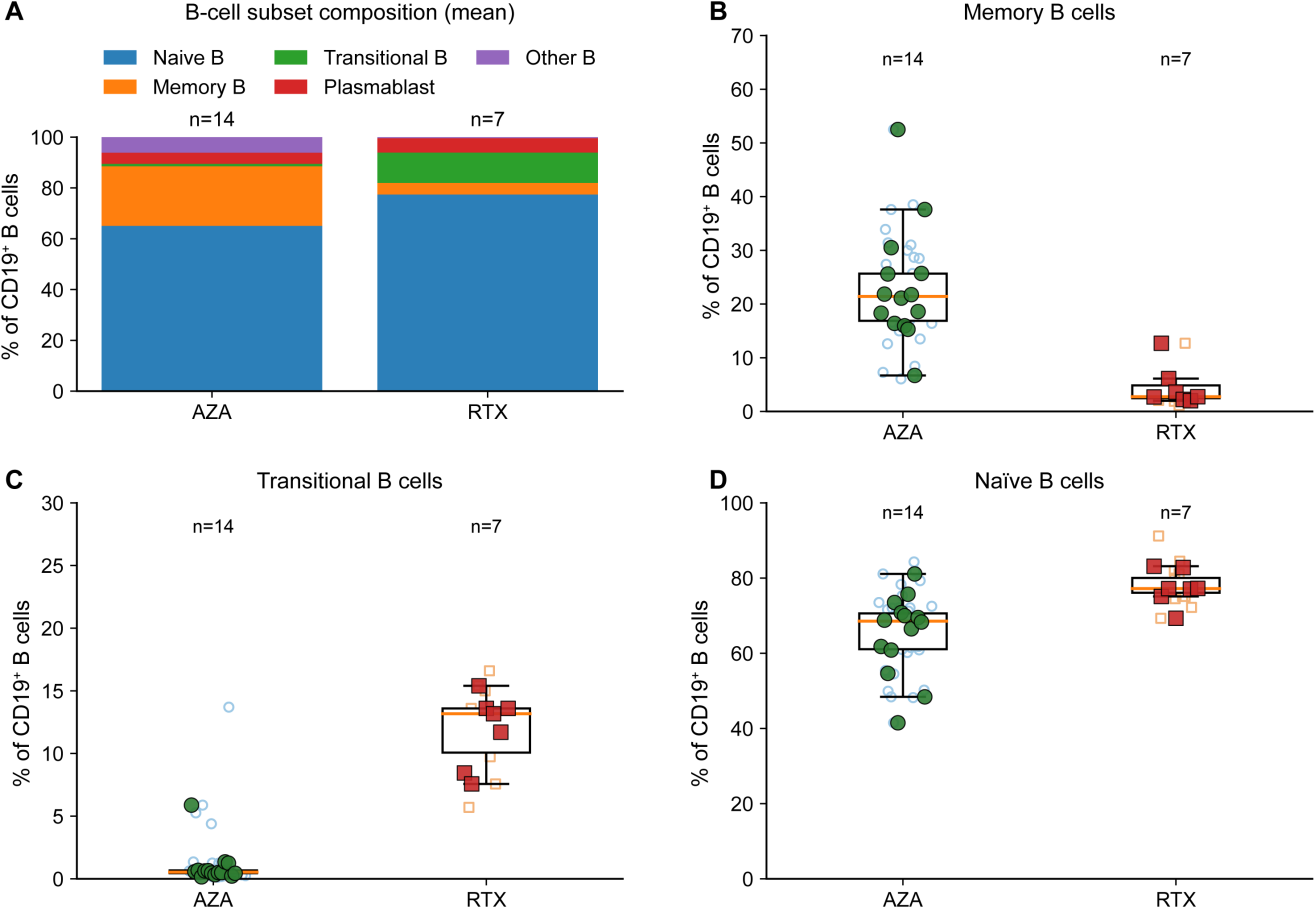
B-cell subset composition in B-detectable samples highlights transitional/naive-skewed reconstitution with memory B-cell suppression under RTX. Because RTX causes profound B-cell depletion, B-cell subset analyses are restricted to B-detectable (subset-evaluable) samples; AZA includes all patients (n=14), whereas RTX panels reflect B-detectable RTX patients (n=7). All subsets are expressed as % of CD19^+^ B cells. **(A)** Mean B-cell subset composition (stacked bars) including naive B, memory B, transitional B, plasmablasts, and residual/other **(B)** Memory B cells. **(C)** Transitional B cells. **(D)** Naive B cells. Light symbols represent individual remission samples and filled symbols represent patient-level medians; boxplots are defined as in **Figure 4**.

Because RTX profoundly suppresses CD19^+^ B cells, B-cell subset phenotyping (naive, memory, transitional, plasmablast) was only interpretable in B-detectable samples. Across the cohort, B-subset data were available in 45 samples (AZA 34 samples from 14 patients; RTX 11 samples from 7 patients). Accordingly, subset analyses reflect composition within the recovered or residual B-cell compartment rather than the entire cohort-wide B-cell pool. Within evaluable samples, RTX showed a striking shift toward transitional and naive B-cell enrichment with a reciprocal depletion of memory B cells (**Figures 5A–D**). In age-adjusted patient-clustered models restricted to evaluable samples, transitional B cells were higher in RTX (ΔRTX-AZA = +10.66 percentage points, 95% CI 8.52 to 12.80; p = 1.33 × 10^-22^; FDR q = 3.18 × 10^-21^; **Supplementary Table 1A**), whereas memory B cells were profoundly reduced (ΔRTX-AZA = -17.78 percentage points, 95% CI -24.10 to -11.45; p = 3.64 × 10^-8^; FDR q = 4.37 × 10^-7^; **Supplementary Table 1A**). Naive B cells were modestly higher under RTX (ΔRTX-AZA = +10.97 percentage points, 95% CI 4.70 to 17.24; p = 6.03 × 10^-4^; FDR q = 0.00289; **Supplementary Table 1A**). Plasmablast proportions did not differ significantly after adjustment (**Supplementary Table 1A**). Under RTX, the likelihood that a remission sample was B-detectable increased with time after infusion (**Supplementary Figure 2**; **Supplementary Table 5**), consistent with a two-stage reconstitution model in which B cells first become detectable and, once detectable, display a transitional/naive-skewed composition (**Figures 5A–D**).

### Conventional T-cell differentiation and helper polarization under RTX

Naive and memory T-cell subsets were quantified as proportions within CD4 or CD8 T cells (representative gating shown in **Supplementary Figure 1**). Descriptively, RTX samples showed higher naive fractions and lower memory fractions, particularly in CD8 T cells (**Supplementary Figures 3A–D**). In age-adjusted patient-clustered models, however, these differences did not survive multiplicity control across the prespecified flow-cytometry panel (**Supplementary Table 1A**). Helper T-cell polarization (Th1, Th17, Th1/Th17, Th2; as proportions within conventional CD4) showed directional but attenuated differences, with the strongest trend for lower Th1/Th17 under RTX that did not remain significant after FDR correction within the same flow panel (**Supplementary Figure 4A**; **Supplementary Table 1A**).

### Regulatory remodeling under RTX: memory-skewed Tregs and lower Th1/Th17-to-Treg balance

**Figure 6:**
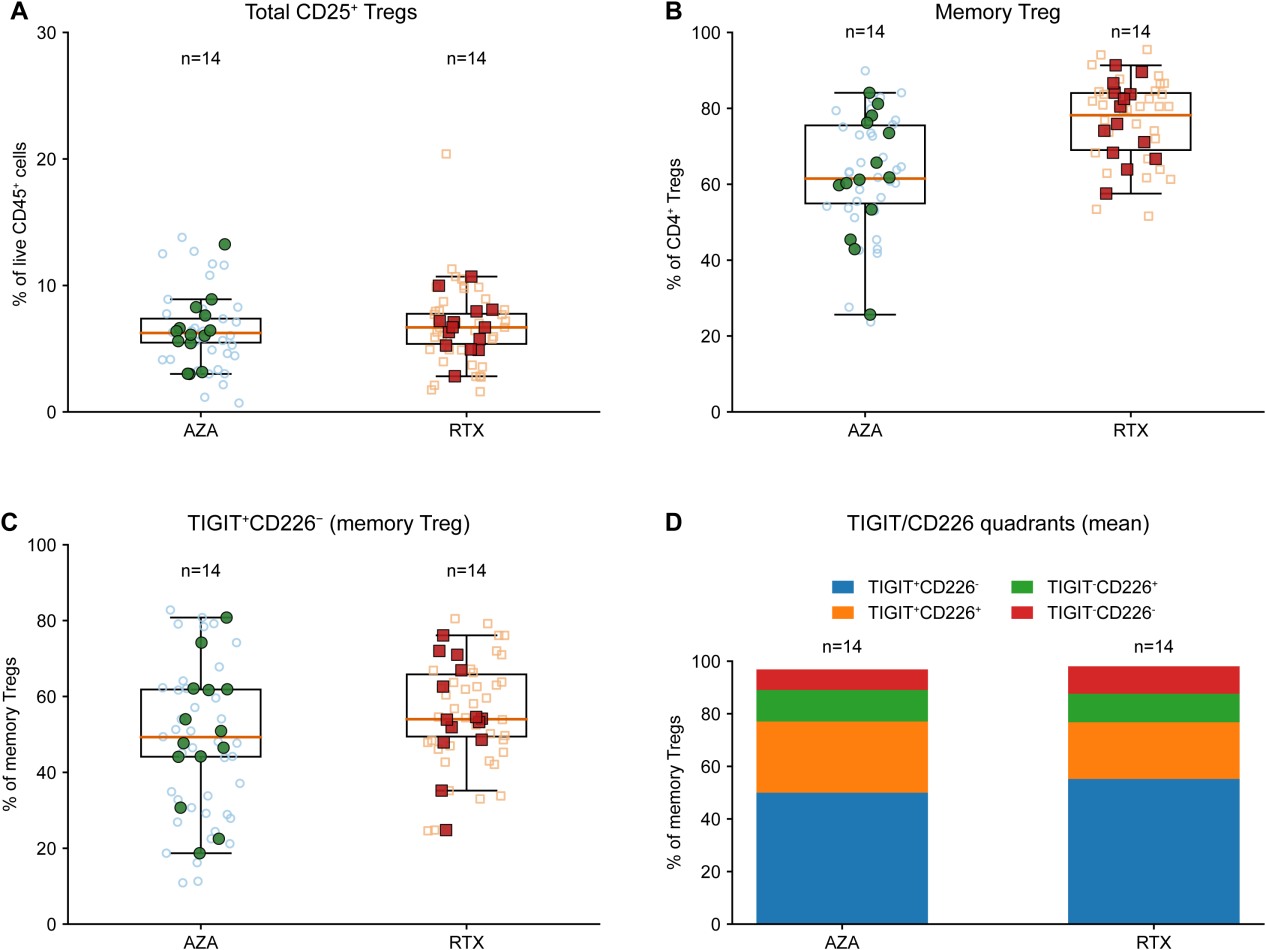
Regulatory compartment remodeling under RTX and TIGIT/CD226 phenotyping. **(A)** Total CD25+ Tregs as % of live CD45^+^ leukocytes. **(B)** Memory Tregs (CD45RO^+^) expressed as % of CD4^+^ Tregs. **(C)** TIGIT^+^CD226^-^ cells expressed as % of memory Tregs. **(D)** Mean TIGIT/CD226 quadrant composition within memory Tregs (stacked bars: TIGIT^+^CD226^-^, TIGIT^+^CD226^+^, TIGIT^-^CD226^+^, TIGIT^-^CD226^-^). Light symbols represent individual remission samples and filled symbols represent patient-level medians; boxplots are defined as in **Figure 4**. n indicates the number of patients per group.

Although the overall frequency of total CD25^+^ Tregs among live CD45^+^ cells was similar in both groups (**Figure 6A**) and not significantly different in adjusted models (**Supplementary Table 1A**), the phenotypic composition of the Treg compartment differed substantially. RTX was associated with higher memory Treg proportions (as % of CD4^+^ Tregs; **Figure 6B**) and lower naive Treg proportions (**Supplementary Table 1A**). In age-adjusted patient-clustered models, memory Treg was higher under RTX (ΔRTX-AZA = +16.42 percentage points, 95% CI 6.16 to 26.68; p = 0.00171; FDR q = 0.00683; **Supplementary Table 1A**), whereas naive Treg was lower (ΔRTX-AZA = -14.97 percentage points, 95% CI -25.00 to -4.94; p = 0.00344; FDR q = 0.0118; **Supplementary Table 1A**). To integrate effector and regulatory axes, a composite Th1/Th17-to-Treg balance index was calculated as log2(Th1/Th17 [% of conventional CD4]/total Treg [% of CD45]) (**Supplementary Figure 4C**). This index was lower under RTX (ΔRTX-AZA = -1.26, 95% CI -2.08 to -0.44; p = 0.00261; **Supplementary Figure 4C**; **Supplementary Table 1A**), consistent with a more Treg-dominant helper-regulatory balance. TIGIT/CD226 quadrants within memory Treg were evaluated as an additional layer of regulatory specialization (**Figures 6C, D**). The TIGIT^+^CD226^-^ fraction showed a trend toward higher values under RTX (**Figure 6C**), but this did not reach significance after multiplicity correction (**Supplementary Table 1A**). The remaining quadrants were not significantly different between therapies (**Supplementary Table 1A**).

### Sensitivity analyses using patient-level medians support robustness of key RTX-associated signatures

Sensitivity analyses based on patient-level medians supported robustness of the major RTX-associated shifts observed in serial-sample models. The most consistent findings across analytic approaches were profound CD19^+^ B-cell depletion, sustained reduction of NKT-like cells, and regulatory remodeling characterized by memory-skewed Tregs and a lower Th1/Th17-to-Treg balance index (**Supplementary Table 2A**). Evidence for time-dependent B-cell reconstitution was derived from RTX-only months-after-infusion models (**Supplementary Table 1C**). Conventional T-cell naive/memory shifts and Th polarization differences were directionally consistent in descriptive comparisons but were more sensitive to adjustment choices and missing subset data (**Supplementary Table 2A**). Cytokine-only sensitivity analyses are provided separately in **Supplementary Table 2B**.

### Therapy-specific cytokine–cell coupling structures during remission

To test whether the two maintenance therapies generate distinct immune network architectures during remission, cytokine–cell coupling was quantified within each treatment group using age-adjusted, patient-clustered models of log10(cytokine + 1) as a function of key immune subsets (Total B, Transitional B, NKT-like, TIGIT^+^CD226^-^ memory Treg). Figure-level results are summarized in **Figure 2**, with complete model outputs in **Supplementary Table 3A**, **S3B**. FDR q values were computed within each treatment across all 48 prespecified cytokine–cell edges (4 predictors × 12 cytokines; **Supplementary Table 3A**, **S3B**). In AZA, the coupling network was dominated by a transitional B-centered module: transitional B fraction (as % of CD19^+^ B) showed a strong positive association with IP-10/CXCL10 (β = +0.151 log10 units per 10 percentage-point increase, 95% CI 0.116 to 0.186; FDR q = 1.24 × 10^-15^) and inverse associations with multiple cytokines including IL-2, IL-4, IL-1β, IL-13, and IL-17A (**Figure 2A**; **Supplementary Table 3A**). Total B cells (as % of CD45^+^) were positively associated with IL-10 in AZA (**Figure 2A**; **Supplementary Table 3A**). In RTX, coupling patterns were qualitatively different and comparatively concentrated on residual B-cell burden and TIGIT-linked regulatory features. Total B cells were positively associated with IFN-γ (β = +0.453 log10 per 10 percentage-point increase, 95% CI 0.252 to 0.653; FDR q = 1.56 × 10^-4^) and with IL-4 (FDR q = 0.0479) (**Figure 2B**; **Supplementary Table 3B**). Separately, TIGIT^+^CD226^-^ within memory Treg showed positive coupling with IL-10 (β = +0.0450 log10 per 10 percentage-point increase, 95% CI 0.0198 to 0.0701; FDR q = 0.00559) and with IL-1β (FDR q = 0.0479) (**Figure 2B**; **Supplementary Table 3B**). In RTX, transitional B-cell analyses were limited to B-detectable samples and therefore reflect conditional reconstitution-state associations rather than cohort-wide treatment effects (**Supplementary Table 3B**).

### Disability-associated immune axes differ by therapy and are supported by treatment × EDSS interaction

Clinical disability coupling was assessed using age-adjusted, patient-clustered models. Standardized coefficients were obtained by z-scoring EDSS and each immune marker within each treatment group; β therefore represents the SD change in the immune marker per 1-SD increase in EDSS (**Figure 3**; **Supplementary Table 4A**). Within-treatment q values reflect Benjamini-Hochberg FDR correction across the prespecified EDSS feature set (m = 8) separately for AZA and RTX, and interaction q values reflect FDR correction across the same 8 tests (**Supplementary Table 4B**). In AZA, EDSS was strongly and positively associated with the TIGIT^+^CD226^-^ memory Treg fraction (β = +0.681, 95% CI 0.345 to 1.018; p = 7.3 × 10^-5^; FDR q = 0.000587), and inversely associated with NKT-like cells (β = -0.603, 95% CI -1.061 to -0.145; p = 0.00984; FDR q = 0.0393) (**Figures 3A, B**; **Supplementary Table 4A**). In RTX, EDSS coupling was more strongly associated with soluble cytokines. EDSS was positively associated with IL-6 (β = +0.637, 95% CI 0.153 to 1.121; p = 0.00995; FDR q = 0.0265) and with IP-10/CXCL10 (β = +0.476, 95% CI 0.120 to 0.831; p = 0.00875; FDR q = 0.0265) (**Figures 3A, C, D**; **Supplementary Table 4A**). Transitional B showed a negative association with EDSS in RTX in the B-detectable subset (β = -0.607; p = 0.00326; FDR q = 0.0261), whereas total B cells were not significantly associated (**Supplementary Table 4A**). Direct comparison of EDSS-immune slopes between therapies demonstrated a regimen-dependent axis for the regulatory marker: the treatment × EDSS interaction for TIGIT^+^CD226^-^ was significant (ΔβRTX-AZA = -0.768, 95% CI -1.215 to -0.321; p = 0.000763; FDR q = 0.00611; **Figure 3E**; **Supplementary Table 4B**). A treatment × EDSS interaction for NKT-like cells reached nominal significance (p = 0.024) but did not remain significant after FDR correction (**Supplementary Table 4B**). Additional robustness models are provided in **Supplementary Table 5** and summarized in **Supplementary Figure 5**.

## Discussion

This study demonstrates that mechanistically distinct maintenance therapies impose divergent remission immune architectures in AQP4-IgG-positive NMOSD. In serial remission samples analyzed with patient-clustered, age-adjusted models, RTX was associated with profound CD19^+^ B-cell depletion, time-dependent reconstitution, sustained reduction of NKT-like cells, and memory-skewed regulatory remodeling. Importantly, disability coupled to different immune axes depending on therapy: under AZA, EDSS aligned with a TIGIT-associated regulatory phenotype, whereas under RTX it aligned with soluble inflammatory mediators, particularly IL-6 and IP-10/CXCL10. Together, these findings argue that remission in NMOSD should be understood as a therapy-conditioned biologic state rather than a treatment-independent state.

The clearest evidence of RTX target engagement was profound B-cell depletion together with time-dependent reconstitution. Although RTX targets CD20 rather than CD19 directly, the marked reduction of circulating CD19^+^ B cells provides a practical peripheral readout of depletion, and the positive association between CD19^+^ frequency and months after infusion is consistent with progressive reconstitution between maintenance doses. Because RTX induced profound depletion, B-cell subset phenotyping was only possible in B-detectable samples; within this subset, the recovering compartment was strongly transitional/naive-skewed with marked suppression of memory B cells. Transitional B cells have been linked to regulatory potential and restoration of B-cell tolerance after depletion therapy, including in autoimmune settings and NMOSD (24, 29, 36). Our evaluability analysis supports a two-stage reconstitution framework in RTX: B cells first re-emerge to detectable levels and, once detectable, remain biased toward early differentiation states. By contrast, AZA provides broad antiproliferative immunosuppression rather than selective B-cell hub depletion, consistent with the persistence of memory compartments in our AZA-treated cohort (14). A second robust RTX-associated feature was the reduction of NKT-like cells during remission, without a clear dependence on infusion timing. The mechanism underlying this sustained reduction remains incompletely understood. B cells are well-established CD1d-expressing antigen-presenting cells capable of presenting lipid antigens to invariant natural killer T (iNKT) cells, and bidirectional NKT–B cell interactions depend on both CD1d-restricted lipid antigen presentation and costimulatory signals (37, 38). Depletion of B cells may therefore indirectly reshape innate-like lymphocyte homeostasis through disruption of these interactions, a mechanism further supported by evidence that B cell-mediated CD1d presentation is essential for iNKT cell maintenance *in vivo* (39). Although our flow cytometric definition captures NKT-like cells rather than canonical iNKT cells, these findings may reflect broader alterations in CD1d-restricted innate-like T-cell biology. However, whether reduced NKT-like cell representation under RTX is protective, reflects redistribution between blood and tissues, or contributes to altered immune regulation during NMOSD remission remains to be determined.

Conventional T-cell differentiation and helper polarization showed directional but comparatively modest changes. RTX samples were descriptively naive-skewed, particularly within CD8 T cells, with a trend toward reduced Th1/Th17 representation. This pattern is biologically plausible given the role of B cells in antigen presentation, costimulation, and cytokine-driven T-cell programming. However, these effects were attenuated after accounting for repeated sampling and multiplicity, indicating that conventional T-cell compositional shifts are secondary to the dominant B-cell, NKT-like, and regulatory signatures. Prior work has implicated Tfh/Th17-linked circuits in NMOSD and has shown that B-cell depletion can modulate B-T interactions, including normalization of altered Tfh subset balance after RTX (17, 19–21).

The regulatory compartment provided the clearest evidence of downstream immune remodeling. Total Treg frequency among live CD45^+^ cells was similar between therapies, yet RTX was associated with a pronounced naive-to-memory shift within Tregs and a lower Th1/Th17-to-Treg balance index, consistent with altered regulatory tone rather than numerical expansion. TIGIT/CD226 phenotyping added clinical granularity rather than strong between-treatment separation: although TIGIT^+^CD226^-^ frequency itself was not significantly different between regimens after correction, disability coupling was strongly therapy-dependent. In AZA, higher EDSS aligned with higher TIGIT^+^CD226^-^ memory Tregs, whereas this relationship was absent in RTX and the treatment × EDSS interaction for TIGIT^+^CD226^-^ was significant. Given that TIGIT and CD226 are linked to divergent human Treg phenotypes and suppressive capacity (40, 41) and that regulatory dysfunction has been described in NMOSD (42), this therapy-specific disability coupling highlights the TIGIT axis as a candidate for mechanistic and functional follow-up.

Soluble mediator patterns during remission also differed between RTX and AZA. Most cytokines were low during remission, but IP-10/CXCL10 was selectively higher under RTX in age-adjusted, patient-clustered models and remained higher after additional adjustment for major cellular shifts, indicating that the RTX-associated IP-10 elevation is not fully explained by these cellular differences. In RTX, disability correlated with IL-6 and IP-10, and these associations persisted in robustness models adjusting for residual B-cell burden or months after infusion, suggesting that a soluble inflammatory axis consistent with innate or interferon-inducible programs remains clinically informative despite profound peripheral B-cell depletion (20, 28, 30). This pattern may reflect compensatory or unmasked activation of such pathways in the context of B-cell depletion. IP-10/CXCL10 is predominantly produced by monocytes, plasmacytoid dendritic cells, and astrocytes, cell types not directly targeted by RTX. B-cell depletion may therefore disinhibit IFN-inducible signaling from these residual innate compartments, consistent with evidence that B cells can modulate pDC-mediated type I IFN responses (43). Alternatively, persistent CNS pathology may sustain glial IP-10 production independently of peripheral B-cell activity, a possibility that warrants further investigation (44, 45).

Cytokine–cell coupling analyses showed that remission was organized into different coupling modules under AZA and RTX. In AZA, a transitional B-centered module linked the transitional fraction to multiple cytokines, including strong positive coupling with IP-10 and inverse coupling with IL-2, IL-4, IL-1β, IL-13, and IL-17A. In RTX, coupling was concentrated on residual B-cell burden (total B with IFNγ and IL-4) and TIGIT-linked regulatory features (TIGIT^+^CD226^-^ memory Tregs with IL-10 and IL-1β). Associations involving transitional B within RTX were restricted to B-detectable samples and should therefore be interpreted as conditional on reconstitution rather than as a cohort-wide property. These topology differences indicate that AZA and RTX sustain remission through distinct immune coupling architectures.

The disability analyses provide a clinically relevant anchor for interpreting these immune states. Under AZA, disability aligned with cellular and regulatory features, most notably TIGIT^+^CD226^-^ memory Tregs and lower NKT-like cells, whereas under RTX, disability aligned primarily with soluble mediators, particularly IL-6 and IP-10, and only secondarily with conditional B-subset composition. The significant treatment × EDSS interaction for TIGIT^+^CD226^-^ indicates that the same immune marker can carry different clinical meaning depending on therapy context, underscoring the need for treatment-aware biomarkers. Clinically, RTX has generally shown stronger relapse prevention than AZA in comparative studies and meta-analyses, although effect sizes vary (12, 13). Our data suggest that this therapeutic profile is accompanied by transitional/naive-skewed reconstitution, regulatory remodeling, and regimen-specific immune-disability coupling.

These findings have translational implications. Peripheral CD19^+^ B-cell recovery and time after infusion provide a quantitative readout of target engagement that can be integrated with clinical scheduling, while the conditional composition of reconstituting B cells may add immune-state resolution. Regulatory remodeling metrics, including memory Treg proportion and the Th1/Th17-to-Treg balance index, together with therapy-specific soluble mediators such as IP-10 and IL-6 under RTX, may inform immune monitoring. However, these candidate markers require prospective validation and should be interpreted in the context of the therapy received.

The CD19-centered immunophenotyping framework employed in this study may provide a useful reference for future monitoring strategies under CD19-targeted therapies such as inebilizumab. However, direct translation of CD19-based readouts to this setting requires caution: because CD19 itself becomes the therapeutic target, its surface detection may be affected by mechanisms such as epitope masking or antigen modulation, potentially confounding quantitative assessment (46). In such contexts, alternative B-cell lineage markers not subject to therapeutic targeting, such as CD22 (47), may serve as more reliable anchors for immunophenotypic monitoring.

This study has limitations. The cohort size was modest and derived from a single center, and treatment assignment was not randomized. Baseline age differed between treatment groups, and although we adjusted for age, residual confounding may remain. In particular, the older age of the AZA-treated group may partially contribute to the relative preservation of memory-associated immune phenotypes, potentially reflecting age-related immunosenescence (48). This should be considered when interpreting between-group differences. Treatment selection may reflect clinical practice patterns as well as underlying disease characteristics, both of which could influence observed immune profiles. The present analysis was restricted to patients receiving RTX and AZA, which together represented the majority of maintenance therapies in our cohort. However, other commonly used agents, such as MMF, were not included due to limited availability of longitudinal PBMC samples, which may limit the generalizability of our findings. Future studies incorporating additional treatment groups, including MMF, would help clarify whether the therapy-conditioned immune architecture identified here extends to other maintenance strategies used in NMOSD. Sampling intervals and timing relative to RTX infusion varied, and B-cell subset phenotyping under RTX was only feasible in a subset of B-detectable samples, reducing the effective sample size for subset-level analyses. Importantly, these analyses were restricted to samples with detectable B-cell reconstitution and may therefore not fully represent the broader RTX-treated population, particularly those with sustained depletion. Accordingly, B-cell subset findings in the RTX group should be interpreted with caution. Our coupling analyses quantify adjusted associations rather than causal relationships, and peripheral blood may not fully capture CNS-compartment immune processes.

Despite these limitations, multiple analytic layers converged on a coherent, therapy-specific model of remission immune organization. RTX-treated remission was characterized by profound B-cell depletion with time-dependent recovery, sustained reduction of NKT-like cells, and memory-skewed regulatory remodeling, together with a soluble mediator and disability-coupling axis centered on IL-6 and IP-10. In contrast, AZA-treated remission preserved a transitional B-centered cytokine coupling module and a TIGIT-linked EDSS axis. Importantly, these findings extend beyond individual immune alterations to define therapy-specific immune architectures that link peripheral immune states to clinical disability. These results position remission-phase immune architecture as a treatment-conditioned property in NMOSD and provide a systems-level framework for therapy-aware biomarker development and longitudinal outcome studies. They further suggest that immune monitoring strategies should be tailored to the treatment context, with greater emphasis on soluble inflammatory mediators in RTX-treated patients and on regulatory T cell–associated phenotypes in AZA-treated patients.

## Data Availability

The original contributions presented in the study are included in the article/**Supplementary Material**. Further inquiries can be directed to the corresponding authors.
