# Supplementary material for: Distinct remission immune architectures under rituximab and azathioprine in AQP4-IgG-positive neuromyelitis optica spectrum disorder
Source: Front Immunol. 2026 May 8;17:1834992. doi: 10.3389/fimmu.2026.1834992 (PMC13194602; doi:10.3389/fimmu.2026.1834992)
Supplement: Supplementary file 1 [file Table1.docx]

**Supplementary Tables**

**Table S1.** Age-adjusted, patient-clustered between-treatment effects (RTX vs AZA) for flow cytometry (24 outcomes), cytokines (12 outcomes), and RTX-only months-after-infusion slopes (24 outcomes). Benjamini-Hochberg FDR was controlled separately within each panel (24 / 12 / 24 tests).

**Table S1A. Flow cytometry (between-treatment effects)**

| **Outcome** | **Denominator** | **AZA median [IQR] (patient)** | **RTX median [IQR] (patient)** | **Adj Δ (RTX-AZA)** | **95% CI low** | **95% CI high** | **p** | **q** | **n_patients** | **n_samples** |
| --- | --- | --- | --- | --- | --- | --- | --- | --- | --- | --- |
| CD19^+^ B | % of live CD45^+^ | 6.02 [5.32-9.64] | 0.12 [0.06-1.14] | -6.983 | -9.634 | -4.333 | 2.42E-07 | 1.94E-06 | 28 | 80 |
| CD3^+^ T | % of live CD45^+^ | 57.50 [49.20-61.94] | 50.05 [45.40-53.27] | -5.566 | -13.012 | 1.879 | 0.143 | 0.264 | 28 | 78 |
| CD25^+^TREG | % of live CD45^+^ | 6.24 [5.48-7.37] | 6.69 [5.37-7.76] | 0.061 | -1.701 | 1.822 | 0.946 | 0.946 | 28 | 77 |
| NKT | % of live CD45^+^ | 4.98 [2.88-9.34] | 1.77 [1.42-2.73] | -4.920 | -7.613 | -2.226 | 3.44E-04 | 0.002 | 27 | 80 |
| NK | % of live CD45^+^ | 11.20 [5.46-17.52] | 12.60 [7.51-14.10] | -0.681 | -5.667 | 4.306 | 0.789 | 0.823 | 27 | 80 |
| Monocyte | % of live CD45^+^ | 19.10 [16.12-24.43] | 24.00 [20.00-28.40] | 1.564 | -3.048 | 6.175 | 0.506 | 0.579 | 27 | 77 |
| Naive CD4^+^ T | % of CD4 T | 42.20 [18.35-52.18] | 50.75 [43.64-61.21] | 6.415 | -6.979 | 19.808 | 0.348 | 0.466 | 26 | 63 |
| Naive CD8^+^ T | % of CD8 T | 6.66 [2.97-16.84] | 21.98 [17.42-41.04] | 8.534 | -1.046 | 18.114 | 0.081 | 0.176 | 26 | 63 |
| Naive Treg | % of CD4^+^ Treg | 32.30 [22.27-41.96] | 20.48 [14.09-29.68] | -14.970 | -24.999 | -4.940 | 0.003 | 0.012 | 28 | 70 |
| Memory Treg | % of CD4^+^ Treg | 61.50 [54.95-75.53] | 78.20 [69.00-84.04] | 16.420 | 6.160 | 26.679 | 0.002 | 0.007 | 28 | 70 |
| Th17 (Conv CD4) | % of conventional CD4 | 6.53 [3.67-9.17] | 7.03 [3.31-8.22] | -1.119 | -3.553 | 1.315 | 0.367 | 0.466 | 26 | 71 |
| Th1/Th17 (Conv CD4) | % of conventional CD4 | 3.34 [1.74-8.44] | 1.21 [0.84-6.49] | -3.969 | -8.018 | 0.080 | 0.055 | 0.164 | 26 | 71 |
| Th1 (Conv CD4) | % of conventional CD4 | 3.45 [2.50-7.64] | 4.92 [1.40-8.78] | -1.552 | -5.397 | 2.292 | 0.429 | 0.514 | 26 | 73 |
| Th2 (Conv CD4) | % of conventional CD4 | 5.26 [3.65-7.55] | 7.41 [4.60-11.12] | 1.862 | -1.724 | 5.449 | 0.309 | 0.466 | 26 | 73 |
| Naive B | % of CD19^+^ B | 68.55 [61.09-70.60] | 77.20 [76.10-80.03] | 10.969 | 4.702 | 17.237 | 6.03E-04 | 0.003 | 21 | 45 |
| Memory B | % of CD19^+^ B | 21.42 [16.88-25.68] | 2.74 [2.46-4.86] | -17.778 | -24.104 | -11.451 | 3.64E-08 | 4.37E-07 | 21 | 45 |
| Transitional B | % of CD19^+^ B | 0.54 [0.45-0.67] | 13.17 [10.07-13.60] | 10.660 | 8.525 | 12.796 | 1.33E-22 | 3.18E-21 | 21 | 45 |
| Plasmabalst | % of CD19^+^ B | 3.52 [2.09-4.26] | 5.58 [2.83-7.32] | 1.396 | -1.649 | 4.442 | 0.369 | 0.466 | 21 | 45 |
| TIGIT^+^CD226^-^ | % of memory Treg | 49.30 [44.12-61.85] | 54.03 [49.42-65.83] | 8.811 | -1.691 | 19.314 | 0.100 | 0.200 | 28 | 81 |
| TIGIT^+^CD226^+^ | % of memory Treg | 22.55 [20.88-27.70] | 21.98 [14.85-27.40] | -5.661 | -11.887 | 0.565 | 0.075 | 0.176 | 28 | 81 |
| TIGIT^-^CD226^+^ | % of memory Treg | 10.11 [3.80-15.85] | 11.25 [4.43-14.70] | -4.358 | -11.037 | 2.322 | 0.201 | 0.345 | 28 | 81 |
| TIGIT^-^CD226^-^ | % of memory Treg | 9.21 [3.72-10.43] | 8.10 [6.58-11.79] | 1.086 | -3.454 | 5.625 | 0.639 | 0.697 | 28 | 81 |
| Memory CD45RO^+^ CD4^+^ T | % of CD4 T | 56.40 [45.08-80.08] | 47.60 [37.31-54.43] | -6.652 | -19.911 | 6.606 | 0.325 | 0.466 | 26 | 63 |
| Memory CD45RO^+^ CD8^+^ T | % of CD8 T | 91.67 [82.64-93.47] | 74.08 [54.78-80.11] | -9.244 | -19.081 | 0.593 | 0.065 | 0.175 | 26 | 63 |

**Table S1B. Cytokines (between-treatment effects)**

| **Cytokine** | **AZA median [IQR] (patient)** | **RTX median [IQR] (patient)** | **Adj Δ log10(x+1) (RTX-AZA)** | **95% CI low** | **95% CI high** | **Fold-change (RTX/AZA)** | **p** | **q** | **n_patients** | **n_samples** |
| --- | --- | --- | --- | --- | --- | --- | --- | --- | --- | --- |
| IL-6 (SIMOA) | 0.97 [0.67-1.38] | 1.31 [1.05-1.70] | 0.086 | -0.014 | 0.185 | 1.218 | 0.093 | 0.371 | 28 | 82 |
| IL-10 (SIMOA) | 7.33 [6.25-10.21] | 5.81 [4.04-7.30] | -0.119 | -0.236 | -9.01E-04 | 0.761 | 0.048 | 0.290 | 28 | 83 |
| GM-CSF | 0.10 [0.00-4.00] | 0.01 [0.00-1.82] | 0.068 | -0.452 | 0.589 | 1.171 | 0.796 | 0.905 | 28 | 83 |
| IFNr | 3.02 [2.02-6.27] | 2.45 [1.32-8.40] | -0.104 | -0.379 | 0.172 | 0.787 | 0.460 | 0.690 | 28 | 83 |
| IL-1b | 1.46 [0.75-2.97] | 1.03 [0.64-2.29] | -0.164 | -0.458 | 0.131 | 0.686 | 0.276 | 0.602 | 28 | 83 |
| IL-2 | 0.19 [0.17-0.66] | 0.35 [0.31-0.55] | -0.066 | -0.292 | 0.160 | 0.859 | 0.566 | 0.755 | 28 | 83 |
| IL-4 | 0.63 [0.42-0.92] | 0.64 [0.59-0.77] | -0.101 | -0.292 | 0.090 | 0.793 | 0.301 | 0.602 | 28 | 83 |
| IL-8 | 6.31 [4.97-7.86] | 6.51 [5.14-7.87] | 0.156 | -0.119 | 0.431 | 1.433 | 0.266 | 0.602 | 28 | 83 |
| IL-13 | 7.54 [3.62-18.33] | 9.12 [3.38-18.07] | -0.177 | -0.593 | 0.240 | 0.666 | 0.406 | 0.690 | 28 | 83 |
| IL-17A | 0.50 [0.30-0.82] | 0.64 [0.45-1.17] | 0.011 | -0.175 | 0.198 | 1.026 | 0.905 | 0.905 | 28 | 83 |
| IP-10 | 263.38 [207.49-314.05] | 396.29 [318.17-512.80] | 0.171 | 0.059 | 0.283 | 1.483 | 0.003 | 0.033 | 28 | 83 |
| TNFa | 23.80 [17.87-29.34] | 25.18 [23.21-28.66] | 0.009 | -0.121 | 0.138 | 1.020 | 0.897 | 0.905 | 28 | 83 |

**Table S1C. RTX-only months-after-infusion slopes**

| **Outcome** | **Denominator** | **Slope per month** | **95% CI low** | **95% CI high** | **p** | **q** | **n_patients** | **n_samples** |
| --- | --- | --- | --- | --- | --- | --- | --- | --- |
| CD19^+^ B | % of live CD45^+^ | 0.438 | 0.080 | 0.796 | 0.017 | 0.397 | 14 | 41 |
| CD3^+^ T | % of live CD45^+^ | -0.378 | -1.479 | 0.723 | 0.501 | 0.924 | 14 | 41 |
| CD25+TREG | % of live CD45^+^ | -0.168 | -0.403 | 0.067 | 0.161 | 0.924 | 14 | 40 |
| NKT | % of live CD45^+^ | 0.057 | -0.029 | 0.144 | 0.195 | 0.924 | 13 | 38 |
| NK | % of live CD45^+^ | -0.139 | -0.503 | 0.224 | 0.453 | 0.924 | 13 | 38 |
| Monocyte | % of live CD45^+^ | 0.104 | -0.518 | 0.727 | 0.743 | 0.944 | 13 | 36 |
| Naive CD4^+^ T | % of CD4 T | -0.026 | -1.418 | 1.365 | 0.970 | 0.970 | 12 | 26 |
| Naive CD8^+^ T | % of CD8 T | 0.300 | -1.007 | 1.607 | 0.653 | 0.944 | 12 | 26 |
| Naive Treg | % of CD4^+^ Treg | -0.411 | -1.334 | 0.512 | 0.383 | 0.924 | 14 | 33 |
| Memory Treg | % of CD4^+^ Treg | 0.461 | -0.507 | 1.429 | 0.351 | 0.924 | 14 | 33 |
| Th17 (Conv CD4) | % of conventional CD4 | 0.133 | -0.321 | 0.587 | 0.565 | 0.940 | 12 | 33 |
| Th1/Th17 (Conv CD4) | % of conventional CD4 | -0.148 | -0.382 | 0.085 | 0.213 | 0.924 | 12 | 33 |
| Th1 (Conv CD4) | % of conventional CD4 | -0.096 | -0.339 | 0.146 | 0.437 | 0.924 | 12 | 35 |
| Th2 (Conv CD4) | % of conventional CD4 | -0.096 | -0.440 | 0.249 | 0.587 | 0.940 | 12 | 35 |
| Naive B | % of CD19^+^ B | 0.376 | -0.366 | 1.119 | 0.321 | 0.924 | 7 | 11 |
| Memory B | % of CD19^+^ B | -0.175 | -0.555 | 0.206 | 0.368 | 0.924 | 7 | 11 |
| Transitional B | % of CD19^+^ B | 0.112 | -0.728 | 0.951 | 0.794 | 0.944 | 7 | 11 |
| Plasmabalst | % of CD19^+^ B | -1.562 | -3.545 | 0.422 | 0.123 | 0.924 | 7 | 11 |
| TIGIT^+^CD226^-^ | % of memory Treg | 0.102 | -0.574 | 0.778 | 0.767 | 0.944 | 14 | 39 |
| TIGIT^+^CD226^+^ | % of memory Treg | -0.081 | -0.680 | 0.518 | 0.791 | 0.944 | 14 | 39 |
| TIGIT^-^CD226^+^ | % of memory Treg | 0.043 | -0.365 | 0.450 | 0.837 | 0.944 | 14 | 39 |
| TIGIT^-^CD226^-^ | % of memory Treg | -0.037 | -0.638 | 0.565 | 0.905 | 0.944 | 14 | 39 |
| Memory CD45RO^+^ CD4^+^ T | % of CD4 T | 0.111 | -1.207 | 1.429 | 0.869 | 0.944 | 12 | 26 |
| Memory CD45RO^+^ CD8^+^ T | % of CD8 T | -0.419 | -1.602 | 0.764 | 0.488 | 0.924 | 12 | 26 |

**Table S2.** Sensitivity analyses using patient-level medians (one value per patient) for the same flow panel (24 outcomes) and cytokine panel (12 outcomes). Benjamini-Hochberg FDR was controlled within each panel (24 and 12 tests).

**Table S2A. Flow cytometry sensitivity (patient-level medians)**

| **Outcome** | **Denominator** | **AZA median [IQR]** | **RTX median [IQR]** | **Δ medians (RTX-AZA)** | **p (MWU)** | **q** | **n_AZA_pat** | **n_RTX_pat** |
| --- | --- | --- | --- | --- | --- | --- | --- | --- |
| CD19^+^ B | % of live CD45^+^ | 6.02 [5.32-9.64] | 0.12 [0.06-1.14] | -5.890 | 1.81E-04 | 0.001 | 14 | 14 |
| CD3^+^ T | % of live CD45^+^ | 57.50 [49.20-61.94] | 50.05 [45.40-53.27] | -7.450 | 0.098 | 0.235 | 14 | 14 |
| CD25^+^TREG | % of live CD45^+^ | 6.24 [5.48-7.37] | 6.69 [5.37-7.76] | 0.445 | 0.629 | 0.795 | 14 | 14 |
| NKT | % of live CD45^+^ | 4.98 [2.88-9.34] | 1.77 [1.42-2.73] | -3.215 | 0.002 | 0.010 | 14 | 13 |
| NK | % of live CD45^+^ | 11.20 [5.46-17.52] | 12.60 [7.51-14.10] | 1.405 | 0.808 | 0.882 | 14 | 13 |
| Monocyte | % of live CD45^+^ | 19.10 [16.12-24.43] | 24.00 [20.00-28.40] | 4.900 | 0.190 | 0.414 | 14 | 13 |
| Naive CD4^+^ T | % of CD4 T | 42.20 [18.35-52.18] | 50.75 [43.64-61.21] | 8.550 | 0.208 | 0.415 | 14 | 12 |
| Naive CD8^+^ T | % of CD8 T | 6.66 [2.97-16.84] | 21.98 [17.42-41.04] | 15.312 | 0.025 | 0.067 | 14 | 12 |
| Naive Treg | % of CD4^+^ Treg | 32.30 [22.27-41.96] | 20.48 [14.09-29.68] | -11.825 | 0.020 | 0.066 | 14 | 14 |
| Memory Treg | % of CD4^+^ Treg | 61.50 [54.95-75.53] | 78.20 [69.00-84.04] | 16.700 | 0.012 | 0.049 | 14 | 14 |
| Th17 (Conv CD4) | % of conventional CD4 | 6.53 [3.67-9.17] | 7.03 [3.31-8.22] | 0.505 | 0.738 | 0.844 | 14 | 12 |
| Th1/Th17 (Conv CD4) | % of conventional CD4 | 3.34 [1.74-8.44] | 1.21 [0.84-6.49] | -2.135 | 0.292 | 0.438 | 14 | 12 |
| Th1 (Conv CD4) | % of conventional CD4 | 3.45 [2.50-7.64] | 4.92 [1.40-8.78] | 1.468 | 0.898 | 0.937 | 14 | 12 |
| Th2 (Conv CD4) | % of conventional CD4 | 5.26 [3.65-7.55] | 7.41 [4.60-11.12] | 2.145 | 0.292 | 0.438 | 14 | 12 |
| Naive B | % of CD19^+^ B | 68.55 [61.09-70.60] | 77.20 [76.10-80.03] | 8.650 | 0.003 | 0.015 | 14 | 7 |
| Memory B | % of CD19^+^ B | 21.42 [16.88-25.68] | 2.74 [2.46-4.86] | -18.685 | 3.44E-05 | 4.13E-04 | 14 | 7 |
| Transitional B | % of CD19^+^ B | 0.54 [0.45-0.67] | 13.17 [10.07-13.60] | 12.635 | 1.72E-05 | 4.13E-04 | 14 | 7 |
| Plasmabalst | % of CD19^+^ B | 3.52 [2.09-4.26] | 5.58 [2.83-7.32] | 2.065 | 0.535 | 0.714 | 14 | 7 |
| TIGIT^+^CD226^-^ | % of memory Treg | 49.30 [44.12-61.85] | 54.03 [49.42-65.83] | 4.725 | 0.280 | 0.438 | 14 | 14 |
| TIGIT^+^CD226^+^ | % of memory Treg | 22.55 [20.88-27.70] | 21.98 [14.85-27.40] | -0.575 | 0.323 | 0.456 | 14 | 14 |
| TIGIT^-^CD226^+^ | % of memory Treg | 10.11 [3.80-15.85] | 11.25 [4.43-14.70] | 1.140 | 0.982 | 0.982 | 14 | 14 |
| TIGIT^-^CD226^-^ | % of memory Treg | 9.21 [3.72-10.43] | 8.10 [6.58-11.79] | -1.115 | 0.730 | 0.844 | 14 | 14 |
| Memory CD45RO+ CD4^+^ T | % of CD4 T | 56.40 [45.08-80.08] | 47.60 [37.31-54.43] | -8.800 | 0.227 | 0.419 | 14 | 12 |
| Memory CD45RO+ CD8^+^ T | % of CD8 T | 91.67 [82.64-93.47] | 74.08 [54.78-80.11] | -17.600 | 0.022 | 0.066 | 14 | 12 |

**Table S2B. Cytokine sensitivity (patient-level medians)**

| **Cytokine** | **AZA median [IQR]** | **RTX median [IQR]** | **Δ medians (RTX-AZA)** | **p (MWU)** | **q** | **n_AZA_pat** | **n_RTX_pat** |
| --- | --- | --- | --- | --- | --- | --- | --- |
| IL-6 (SIMOA) | 0.97 [0.67-1.38] | 1.31 [1.05-1.70] | 0.340 | 0.069 | 0.278 | 14 | 14 |
| IL-10 (SIMOA) | 7.33 [6.25-10.21] | 5.81 [4.04-7.30] | -1.515 | 0.057 | 0.278 | 14 | 14 |
| GM-CSF | 0.10 [0.00-4.00] | 0.01 [0.00-1.82] | -0.080 | 0.888 | 0.888 | 14 | 14 |
| IFNr | 3.02 [2.02-6.27] | 2.45 [1.32-8.40] | -0.575 | 0.421 | 0.842 | 14 | 14 |
| IL-1b | 1.46 [0.75-2.97] | 1.03 [0.64-2.29] | -0.425 | 0.613 | 0.856 | 14 | 14 |
| IL-2 | 0.19 [0.17-0.66] | 0.35 [0.31-0.55] | 0.160 | 0.346 | 0.830 | 14 | 14 |
| IL-4 | 0.63 [0.42-0.92] | 0.64 [0.59-0.77] | 0.010 | 0.713 | 0.856 | 14 | 14 |
| IL-8 | 6.31 [4.97-7.86] | 6.51 [5.14-7.87] | 0.200 | 0.800 | 0.873 | 14 | 14 |
| IL-13 | 7.54 [3.62-18.33] | 9.12 [3.38-18.07] | 1.585 | 0.679 | 0.856 | 14 | 14 |
| IL-17A | 0.50 [0.30-0.82] | 0.64 [0.45-1.17] | 0.140 | 0.334 | 0.830 | 14 | 14 |
| IP-10 | 263.38 [207.49-314.05] | 396.29 [318.17-512.80] | 132.910 | 0.007 | 0.086 | 14 | 14 |
| TNFa | 23.80 [17.87-29.34] | 25.18 [23.21-28.66] | 1.375 | 0.597 | 0.856 | 14 | 14 |

**S3.** Treatment-stratified cytokine-cell coupling models underlying Fig. 5. Within AZA and within RTX, age-adjusted patient-clustered regressions of log10(cytokine+1) on four prespecified predictors scaled per +10 percentage points. Benjamini-Hochberg FDR was controlled within each treatment across 48 edges (4×12). Transitional B in RTX is restricted to B-detectable samples.

**Table S3A. Cytokine-cell coupling models (AZA) (Fig. 5)**

| **Treatment** | **Predictor** | **Cytokine** | **Slope log10(pg/mL+1) per +10pp predictor** | **95% CI low** | **95% CI high** | **p** | **q** | **n_patients** | **n_samples** | **Note** |
| --- | --- | --- | --- | --- | --- | --- | --- | --- | --- | --- |
| AZA | Total B | IL-6 (SIMOA) | -0.015 | -0.068 | 0.039 | 0.588 | 0.672 | 14 | 39 |  |
| AZA | Total B | IL-10 (SIMOA) | 0.069 | 0.034 | 0.103 | 9.67E-05 | 0.001 | 14 | 39 |  |
| AZA | Total B | GM-CSF | -0.063 | -0.285 | 0.159 | 0.580 | 0.672 | 14 | 39 |  |
| AZA | Total B | IFNr | 0.081 | -0.073 | 0.235 | 0.302 | 0.529 | 14 | 39 |  |
| AZA | Total B | IL-1b | 0.055 | -0.030 | 0.139 | 0.205 | 0.448 | 14 | 39 |  |
| AZA | Total B | IL-2 | -0.030 | -0.087 | 0.028 | 0.311 | 0.529 | 14 | 39 |  |
| AZA | Total B | IL-4 | -3.37E-04 | -0.048 | 0.047 | 0.989 | 0.989 | 14 | 39 |  |
| AZA | Total B | IL-8 | 0.116 | -0.027 | 0.258 | 0.111 | 0.381 | 14 | 39 |  |
| AZA | Total B | IL-13 | 0.143 | -0.060 | 0.345 | 0.167 | 0.420 | 14 | 39 |  |
| AZA | Total B | IL-17A | 0.023 | 0.002 | 0.043 | 0.029 | 0.155 | 14 | 39 |  |
| AZA | Total B | IP-10 | -0.024 | -0.101 | 0.052 | 0.533 | 0.640 | 14 | 39 |  |
| AZA | Total B | TNFa | -0.006 | -0.105 | 0.093 | 0.910 | 0.930 | 14 | 39 |  |
| AZA | Transitional B | IL-6 (SIMOA) | -0.071 | -0.155 | 0.013 | 0.099 | 0.364 | 14 | 34 |  |
| AZA | Transitional B | IL-10 (SIMOA) | -0.101 | -0.192 | -0.011 | 0.028 | 0.155 | 14 | 34 |  |
| AZA | Transitional B | GM-CSF | 0.058 | -0.041 | 0.157 | 0.252 | 0.484 | 14 | 34 |  |
| AZA | Transitional B | IFNr | -0.113 | -0.242 | 0.015 | 0.084 | 0.336 | 14 | 34 |  |
| AZA | Transitional B | IL-1b | -0.203 | -0.308 | -0.097 | 1.59E-04 | 0.002 | 14 | 34 |  |
| AZA | Transitional B | IL-2 | -0.188 | -0.267 | -0.108 | 3.65E-06 | 8.77E-05 | 14 | 34 |  |
| AZA | Transitional B | IL-4 | -0.173 | -0.258 | -0.087 | 7.32E-05 | 0.001 | 14 | 34 |  |
| AZA | Transitional B | IL-8 | 0.112 | -0.053 | 0.278 | 0.184 | 0.420 | 14 | 34 |  |
| AZA | Transitional B | IL-13 | -0.205 | -0.326 | -0.084 | 9.11E-04 | 0.007 | 14 | 34 |  |
| AZA | Transitional B | IL-17A | -0.196 | -0.327 | -0.066 | 0.003 | 0.021 | 14 | 34 |  |
| AZA | Transitional B | IP-10 | 0.151 | 0.116 | 0.186 | 2.59E-17 | 1.24E-15 | 14 | 34 |  |
| AZA | Transitional B | TNFa | -0.033 | -0.134 | 0.069 | 0.529 | 0.640 | 14 | 34 |  |
| AZA | NKT | IL-6 (SIMOA) | -0.034 | -0.072 | 0.004 | 0.083 | 0.336 | 14 | 42 |  |
| AZA | NKT | IL-10 (SIMOA) | -0.024 | -0.068 | 0.020 | 0.278 | 0.514 | 14 | 42 |  |
| AZA | NKT | GM-CSF | -0.086 | -0.196 | 0.023 | 0.121 | 0.387 | 14 | 42 |  |
| AZA | NKT | IFNr | -0.028 | -0.146 | 0.090 | 0.643 | 0.701 | 14 | 42 |  |
| AZA | NKT | IL-1b | -0.059 | -0.144 | 0.027 | 0.180 | 0.420 | 14 | 42 |  |
| AZA | NKT | IL-2 | -0.030 | -0.078 | 0.018 | 0.223 | 0.466 | 14 | 42 |  |
| AZA | NKT | IL-4 | -0.009 | -0.036 | 0.018 | 0.503 | 0.640 | 14 | 42 |  |
| AZA | NKT | IL-8 | -0.016 | -0.094 | 0.062 | 0.691 | 0.737 | 14 | 42 |  |
| AZA | NKT | IL-13 | 0.078 | -0.102 | 0.258 | 0.397 | 0.596 | 14 | 42 |  |
| AZA | NKT | IL-17A | 0.024 | -0.008 | 0.056 | 0.136 | 0.409 | 14 | 42 |  |
| AZA | NKT | IP-10 | 0.013 | -0.016 | 0.042 | 0.368 | 0.570 | 14 | 42 |  |
| AZA | NKT | TNFa | 0.018 | -0.028 | 0.064 | 0.440 | 0.621 | 14 | 42 |  |
| AZA | TIGIT^+^CD226^-^ | IL-6 (SIMOA) | -0.003 | -0.023 | 0.018 | 0.811 | 0.846 | 14 | 42 |  |
| AZA | TIGIT^+^CD226^-^ | IL-10 (SIMOA) | -0.005 | -0.019 | 0.009 | 0.477 | 0.640 | 14 | 42 |  |
| AZA | TIGIT^+^CD226^-^ | GM-CSF | 0.025 | -0.025 | 0.074 | 0.334 | 0.534 | 14 | 42 |  |
| AZA | TIGIT^+^CD226^-^ | IFNr | -0.018 | -0.043 | 0.008 | 0.169 | 0.420 | 14 | 42 |  |
| AZA | TIGIT^+^CD226^-^ | IL-1b | 0.013 | -0.012 | 0.037 | 0.319 | 0.529 | 14 | 42 |  |
| AZA | TIGIT^+^CD226^-^ | IL-2 | 0.005 | -0.010 | 0.021 | 0.514 | 0.640 | 14 | 42 |  |
| AZA | TIGIT^+^CD226^-^ | IL-4 | 0.008 | -0.006 | 0.023 | 0.250 | 0.484 | 14 | 42 |  |
| AZA | TIGIT^+^CD226^-^ | IL-8 | -0.033 | -0.081 | 0.014 | 0.164 | 0.420 | 14 | 42 |  |
| AZA | TIGIT^+^CD226^-^ | IL-13 | -0.010 | -0.051 | 0.030 | 0.613 | 0.684 | 14 | 42 |  |
| AZA | TIGIT^+^CD226^-^ | IL-17A | -0.009 | -0.018 | -5.18E-04 | 0.038 | 0.182 | 14 | 42 |  |
| AZA | TIGIT^+^CD226^-^ | IP-10 | 0.006 | -0.013 | 0.026 | 0.515 | 0.640 | 14 | 42 |  |
| AZA | TIGIT^+^CD226^-^ | TNFa | 0.010 | -0.013 | 0.032 | 0.412 | 0.599 | 14 | 42 |  |

**Table S3B. Cytokine-cell coupling models (RTX) (Fig. 5)**

| **Treatment** | **Predictor** | **Cytokine** | **Slope log10(pg/mL+1) per +10pp predictor** | **95% CI low** | **95% CI high** | **p** | **q** | **n_patients** | **n_samples** | **Note** |
| --- | --- | --- | --- | --- | --- | --- | --- | --- | --- | --- |
| RTX | Total B | IL-6 (SIMOA) | 0.107 | -0.074 | 0.287 | 0.245 | 0.491 | 14 | 40 |  |
| RTX | Total B | IL-10 (SIMOA) | 0.052 | -0.111 | 0.215 | 0.534 | 0.754 | 14 | 41 |  |
| RTX | Total B | GM-CSF | -0.190 | -0.365 | -0.015 | 0.033 | 0.151 | 14 | 41 |  |
| RTX | Total B | IFNr | 0.453 | 0.252 | 0.653 | 9.73E-06 | 1.56E-04 | 14 | 41 |  |
| RTX | Total B | IL-1b | 0.259 | -0.120 | 0.637 | 0.180 | 0.412 | 14 | 41 |  |
| RTX | Total B | IL-2 | 0.178 | -0.010 | 0.365 | 0.063 | 0.202 | 14 | 41 |  |
| RTX | Total B | IL-4 | 0.209 | 0.060 | 0.358 | 0.006 | 0.048 | 14 | 41 |  |
| RTX | Total B | IL-8 | 0.332 | -0.212 | 0.877 | 0.231 | 0.491 | 14 | 41 |  |
| RTX | Total B | IL-13 | 0.244 | -0.029 | 0.517 | 0.080 | 0.226 | 14 | 41 |  |
| RTX | Total B | IL-17A | 0.169 | -0.002 | 0.340 | 0.053 | 0.196 | 14 | 41 |  |
| RTX | Total B | IP-10 | 0.046 | -0.031 | 0.123 | 0.241 | 0.491 | 14 | 41 |  |
| RTX | Total B | TNFa | 0.118 | 0.016 | 0.219 | 0.023 | 0.125 | 14 | 41 |  |
| RTX | Transitional B | IL-6 (SIMOA) | -0.134 | -1.208 | 0.940 | 0.807 | 0.901 | 7 | 11 | B-detectable RTX subset (n_patients=7) |
| RTX | Transitional B | IL-10 (SIMOA) | -1.407 | -2.550 | -0.264 | 0.016 | 0.109 | 7 | 11 | B-detectable RTX subset (n_patients=7) |
| RTX | Transitional B | GM-CSF | 0.624 | -0.073 | 1.321 | 0.079 | 0.226 | 7 | 11 | B-detectable RTX subset (n_patients=7) |
| RTX | Transitional B | IFNr | -0.497 | -1.769 | 0.775 | 0.444 | 0.706 | 7 | 11 | B-detectable RTX subset (n_patients=7) |
| RTX | Transitional B | IL-1b | -1.056 | -5.653 | 3.540 | 0.652 | 0.803 | 7 | 11 | B-detectable RTX subset (n_patients=7) |
| RTX | Transitional B | IL-2 | 0.280 | -3.082 | 3.642 | 0.870 | 0.908 | 7 | 11 | B-detectable RTX subset (n_patients=7) |
| RTX | Transitional B | IL-4 | 40.589 | -1.770 | 82.947 | 0.060 | 0.202 | 7 | 11 | B-detectable RTX subset (n_patients=7) |
| RTX | Transitional B | IL-8 | -2.680 | -3.478 | -1.882 | 4.52E-11 | 1.09E-09 | 7 | 11 | B-detectable RTX subset (n_patients=7) |
| RTX | Transitional B | IL-13 | -0.595 | -1.822 | 0.632 | 0.342 | 0.586 | 7 | 11 | B-detectable RTX subset (n_patients=7) |
| RTX | Transitional B | IL-17A | -0.269 | -0.794 | 0.256 | 0.315 | 0.561 | 7 | 11 | B-detectable RTX subset (n_patients=7) |
| RTX | Transitional B | IP-10 | 0.450 | -0.428 | 1.327 | 0.316 | 0.561 | 7 | 11 | B-detectable RTX subset (n_patients=7) |
| RTX | Transitional B | TNFa | -0.513 | -0.653 | -0.373 | 6.91E-13 | 3.32E-11 | 7 | 11 | B-detectable RTX subset (n_patients=7) |
| RTX | NKT | IL-6 (SIMOA) | 0.366 | -0.108 | 0.840 | 0.130 | 0.313 | 13 | 37 |  |
| RTX | NKT | IL-10 (SIMOA) | -0.018 | -0.175 | 0.138 | 0.818 | 0.901 | 13 | 38 |  |
| RTX | NKT | GM-CSF | -0.317 | -1.533 | 0.899 | 0.610 | 0.787 | 13 | 38 |  |
| RTX | NKT | IFNr | -0.224 | -0.816 | 0.367 | 0.457 | 0.706 | 13 | 38 |  |
| RTX | NKT | IL-1b | -0.132 | -0.913 | 0.649 | 0.741 | 0.867 | 13 | 38 |  |
| RTX | NKT | IL-2 | 0.026 | -0.235 | 0.287 | 0.845 | 0.901 | 13 | 38 |  |
| RTX | NKT | IL-4 | 0.070 | -0.181 | 0.321 | 0.586 | 0.787 | 13 | 38 |  |
| RTX | NKT | IL-8 | 0.328 | -0.979 | 1.634 | 0.623 | 0.787 | 13 | 38 |  |
| RTX | NKT | IL-13 | -0.128 | -0.510 | 0.255 | 0.513 | 0.746 | 13 | 38 |  |
| RTX | NKT | IL-17A | 0.005 | -0.266 | 0.276 | 0.972 | 0.972 | 13 | 38 |  |
| RTX | NKT | IP-10 | -0.034 | -0.362 | 0.294 | 0.840 | 0.901 | 13 | 38 |  |
| RTX | NKT | TNFa | -0.014 | -0.280 | 0.252 | 0.919 | 0.939 | 13 | 38 |  |
| RTX | TIGIT^+^CD226^-^ | IL-6 (SIMOA) | 0.006 | -0.030 | 0.043 | 0.733 | 0.867 | 14 | 38 |  |
| RTX | TIGIT^+^CD226^-^ | IL-10 (SIMOA) | 0.045 | 0.020 | 0.070 | 4.66E-04 | 0.006 | 14 | 39 |  |
| RTX | TIGIT^+^CD226^-^ | GM-CSF | 0.170 | 0.028 | 0.312 | 0.019 | 0.112 | 14 | 39 |  |
| RTX | TIGIT^+^CD226^-^ | IFNr | 0.061 | 0.004 | 0.118 | 0.037 | 0.151 | 14 | 39 |  |
| RTX | TIGIT^+^CD226^-^ | IL-1b | 0.134 | 0.039 | 0.230 | 0.006 | 0.048 | 14 | 39 |  |
| RTX | TIGIT^+^CD226^-^ | IL-2 | 0.016 | -0.021 | 0.054 | 0.392 | 0.649 | 14 | 39 |  |
| RTX | TIGIT^+^CD226^-^ | IL-4 | 0.017 | -0.002 | 0.037 | 0.086 | 0.229 | 14 | 39 |  |
| RTX | TIGIT^+^CD226^-^ | IL-8 | 0.178 | 0.010 | 0.347 | 0.038 | 0.151 | 14 | 39 |  |
| RTX | TIGIT^+^CD226^-^ | IL-13 | 0.045 | -0.008 | 0.098 | 0.094 | 0.237 | 14 | 39 |  |
| RTX | TIGIT^+^CD226^-^ | IL-17A | 0.012 | -0.032 | 0.055 | 0.602 | 0.787 | 14 | 39 |  |
| RTX | TIGIT^+^CD226^-^ | IP-10 | 0.019 | -0.015 | 0.054 | 0.268 | 0.515 | 14 | 39 |  |
| RTX | TIGIT^+^CD226^-^ | TNFa | 0.012 | -0.020 | 0.044 | 0.471 | 0.706 | 14 | 39 |  |

**Table S4A.** Within-treatment EDSS coupling models underlying Fig. 6A. Standardized coefficients (beta) from age-adjusted patient-clustered models after z-scoring EDSS and each outcome within each treatment. Benjamini-Hochberg FDR was controlled within treatment across the prespecified EDSS feature set (8 tests per treatment).

**Table S4A. Within-treatment EDSS coupling models (Fig. 6A)**

| **Treatment** | **Outcome** | **β (std)** | **95% CI low** | **95% CI high** | **p** | **q** | **n_patients** | **n_samples** | **Note** |
| --- | --- | --- | --- | --- | --- | --- | --- | --- | --- |
| AZA | Total B | 0.026 | -0.350 | 0.402 | 0.892 | 0.892 | 14 | 39 |  |
| AZA | Transitional B | -0.043 | -0.247 | 0.160 | 0.676 | 0.801 | 14 | 34 |  |
| AZA | NKT | -0.603 | -1.061 | -0.145 | 0.010 | 0.039 | 14 | 42 |  |
| AZA | TIGIT^+^CD226^-^ | 0.681 | 0.345 | 1.018 | 7.34E-05 | 5.87E-04 | 14 | 42 |  |
| AZA | IL-6 (SIMOA) (log10+1) | 0.143 | -0.401 | 0.687 | 0.606 | 0.801 | 14 | 42 |  |
| AZA | IL-13 (log10+1) | -0.201 | -0.675 | 0.274 | 0.407 | 0.801 | 14 | 42 |  |
| AZA | IP-10 (log10+1) | 0.333 | -0.112 | 0.778 | 0.143 | 0.381 | 14 | 42 |  |
| AZA | TNFa (log10+1) | -0.079 | -0.479 | 0.322 | 0.700 | 0.801 | 14 | 42 |  |
| RTX | Total B | 0.301 | -0.170 | 0.772 | 0.211 | 0.281 | 14 | 41 |  |
| RTX | Transitional B | -0.607 | -1.011 | -0.202 | 0.003 | 0.026 | 7 | 11 | B-detectable RTX subset (n_patients=7) |
| RTX | NKT | 0.279 | 0.008 | 0.550 | 0.044 | 0.070 | 13 | 38 |  |
| RTX | TIGIT^+^CD226^-^ | -0.095 | -0.456 | 0.267 | 0.608 | 0.608 | 14 | 39 |  |
| RTX | IL-6 (SIMOA) (log10+1) | 0.637 | 0.153 | 1.121 | 0.010 | 0.027 | 14 | 40 |  |
| RTX | IL-13 (log10+1) | -0.206 | -0.633 | 0.221 | 0.345 | 0.394 | 14 | 41 |  |
| RTX | IP-10 (log10+1) | 0.476 | 0.120 | 0.831 | 0.009 | 0.027 | 14 | 41 |  |
| RTX | TNFa (log10+1) | 0.423 | 0.029 | 0.817 | 0.035 | 0.070 | 14 | 41 |  |

**Table S4B.** Treatment×EDSS interaction models underlying Fig. 6E. Differences in standardized EDSS slopes (Delta beta RTX-AZA) for the same 8-feature set. Benjamini-Hochberg FDR was controlled across 8 interaction tests.

**Table S4B. Treatment×EDSS interaction models (Fig. 6E)**

| **Outcome** | **Slope_AZA (β std)** | **Slope_RTX (β std)** | **Δ slope (RTX-AZA)** | **95% CI low** | **95% CI high** | **p_int** | **q_int** | **n_patients** | **n_samples** | **Note** |
| --- | --- | --- | --- | --- | --- | --- | --- | --- | --- | --- |
| Total B | -0.104 | 0.236 | 0.339 | -0.005 | 0.684 | 0.054 | 0.143 | 28 | 80 |  |
| Transitional B | -0.130 | -0.100 | 0.030 | -0.369 | 0.429 | 0.882 | 0.882 | 21 | 45 | B-detectable subset only (AZA n=14; RTX n=7; total n=21) |
| NKT | -0.537 | -0.071 | 0.466 | 0.061 | 0.872 | 0.024 | 0.096 | 27 | 80 |  |
| TIGIT^+^CD226^-^ | 0.694 | -0.074 | -0.768 | -1.215 | -0.321 | 7.63E-04 | 0.006 | 28 | 81 |  |
| IL-6 (SIMOA) (log10+1) | 0.360 | 0.596 | 0.236 | -0.340 | 0.812 | 0.422 | 0.770 | 28 | 82 |  |
| IL-13 (log10+1) | -0.101 | -0.223 | -0.123 | -0.639 | 0.393 | 0.641 | 0.854 | 28 | 83 |  |
| IP-10 (log10+1) | 0.299 | 0.458 | 0.159 | -0.283 | 0.601 | 0.482 | 0.770 | 28 | 83 |  |
| TNFa (log10+1) | 0.150 | 0.224 | 0.074 | -0.556 | 0.704 | 0.818 | 0.882 | 28 | 83 |  |

**Table S5.** Additional prespecified analyses reported with nominal p-values only (no FDR correction), including IP-10/CXCL10 treatment effects after adjustment for major cellular shifts, RTX EDSS-cytokine models adjusted for residual B burden or months after infusion, and RTX B-subset evaluability (B-detectability) as a function of months after RTX infusion.

**Table S5. Additional prespecified analyses (nominal p-values)**

| **Analysis** | **Outcome** | **Model** | **Effect** | **Estimate (β)** | **95% CI low** | **95% CI high** | **p-value** | **n patients** | **n samples** | **Estimate type** | **OR (if applicable)** | **OR 95% CI** |
| --- | --- | --- | --- | --- | --- | --- | --- | --- | --- | --- | --- | --- |
| Between-treatment (all samples) | IP-10/CXCL10 [log10(x+1)] | log10(IP10+1) ~ Treatment + Age + TotalB + NKT (patient-clustered GEE, exchangeable) | Treatment (RTX vs AZA) | 0.182 | 0.053 | 0.311 | 0.005588 | 27 | 77 | Δ log10 units |  |  |
| Within RTX | IL-6 (SIMOA) [log10(x+1)] (standardized) | z(log10(IL6+1)) ~ z(EDSS) + z(Age) + z(TotalB) (patient-clustered GEE) | EDSS (per 1 SD) | 0.584 | 0.192 | 0.977 | 0.003524 | 14 | 40 | Standardized β |  |  |
| Within RTX | IP-10/CXCL10 [log10(x+1)] (standardized) | z(log10(IP10+1)) ~ z(EDSS) + z(Age) + z(TotalB) (patient-clustered GEE) | EDSS (per 1 SD) | 0.460 | 0.115 | 0.805 | 0.009044 | 14 | 41 | Standardized β |  |  |
| Within RTX | IL-6 (SIMOA) [log10(x+1)] (standardized) | z(log10(IL6+1)) ~ z(EDSS) + z(Age) + z(Months after RTX) (patient-clustered GEE) | EDSS (per 1 SD) | 0.598 | 0.136 | 1.060 | 0.01115 | 14 | 40 | Standardized β |  |  |
| Within RTX | IP-10/CXCL10 [log10(x+1)] (standardized) | z(log10(IP10+1)) ~ z(EDSS) + z(Age) + z(Months after RTX) (patient-clustered GEE) | EDSS (per 1 SD) | 0.495 | 0.126 | 0.865 | 0.008631 | 14 | 41 | Standardized β |  |  |
| RTX B-detectability (two-stage model) | B subset evaluable (1=yes; 0=no) | logit(Bdetect) ~ Months after RTX + Age (patient-clustered GEE, binomial) | Months after RTX (per 1 month) | 0.328 | 0.048 | 0.608 | 0.02186 | 14 | 41 | Log-odds (β) | 1.388 | 1.05 to 1.84 |
