# Supplementary material for: Distinct remission immune architectures under rituximab and azathioprine in AQP4-IgG-positive neuromyelitis optica spectrum disorder
Source: Front Immunol. 2026 May 8;17:1834992. doi: 10.3389/fimmu.2026.1834992 (PMC13194602; doi:10.3389/fimmu.2026.1834992)
Supplement: Supplementary file 2 [file Image1.pdf]

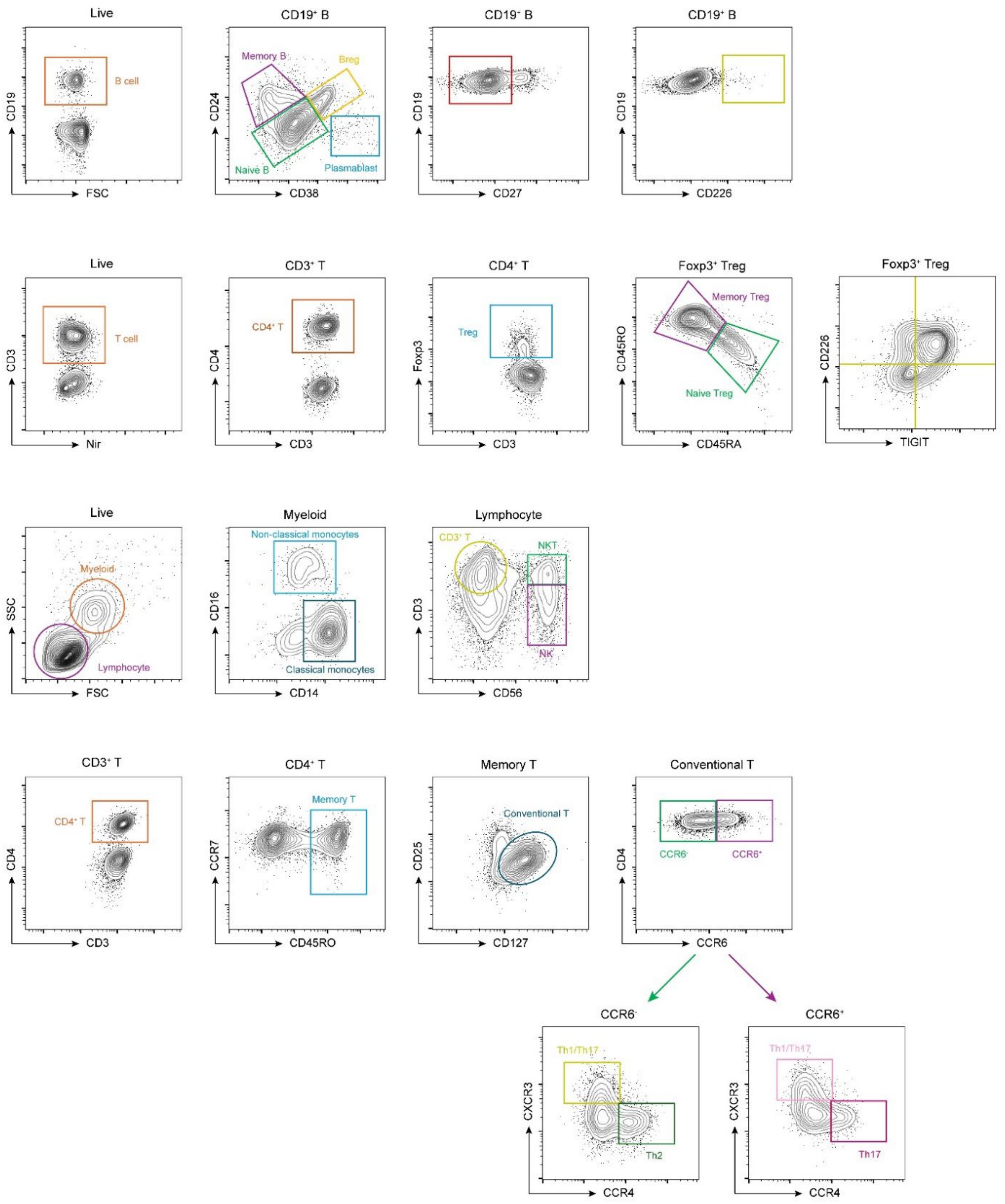

Figure S1. Representative flow-cytometry gating strategy

Sequential gating identified live CD45$^+$ leukocytes and major immune populations, including CD19$^+$ B cells, CD3$^+$ T cells, NK cells, NKT-like cells, and monocytes. Downstream gates defined B-cell

subsets, naive and memory Tregs, TIGIT/CD226 quadrants within memory Tregs, and helper T-cell subsets used for subsequent analyses.

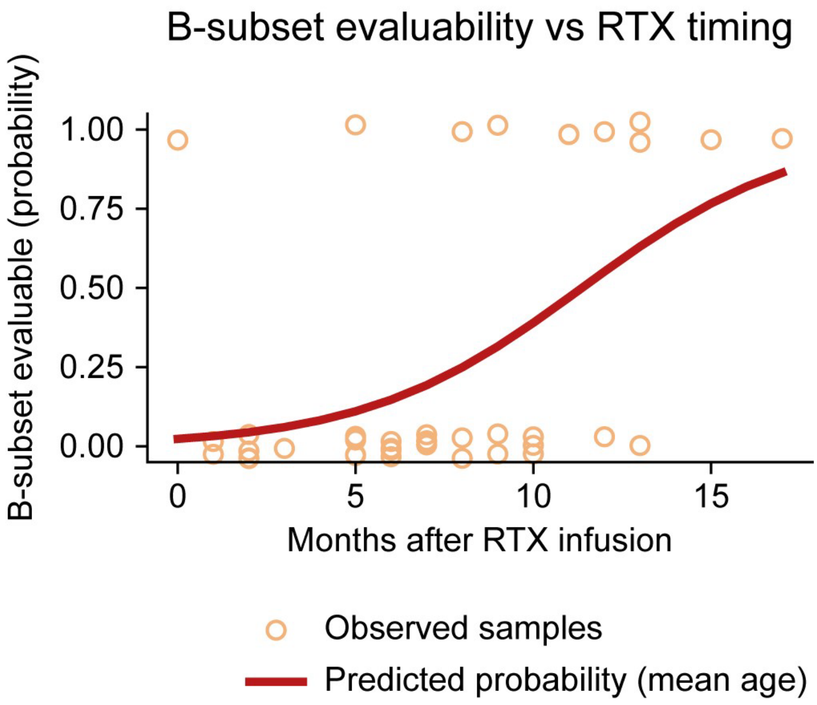

Figure S2. Two-stage RTX B-cell reconstitution

RTX-only analysis evaluating whether the probability that a sample is B-subset evaluable (B-detectable) increases with months after the most recent RTX infusion. Each open circle represents an observed remission sample classified as evaluable or non-evaluable for B-subset gating. The red curve indicates the predicted probability of evaluability from an age-adjusted, patient-clustered binomial model, shown at mean age.

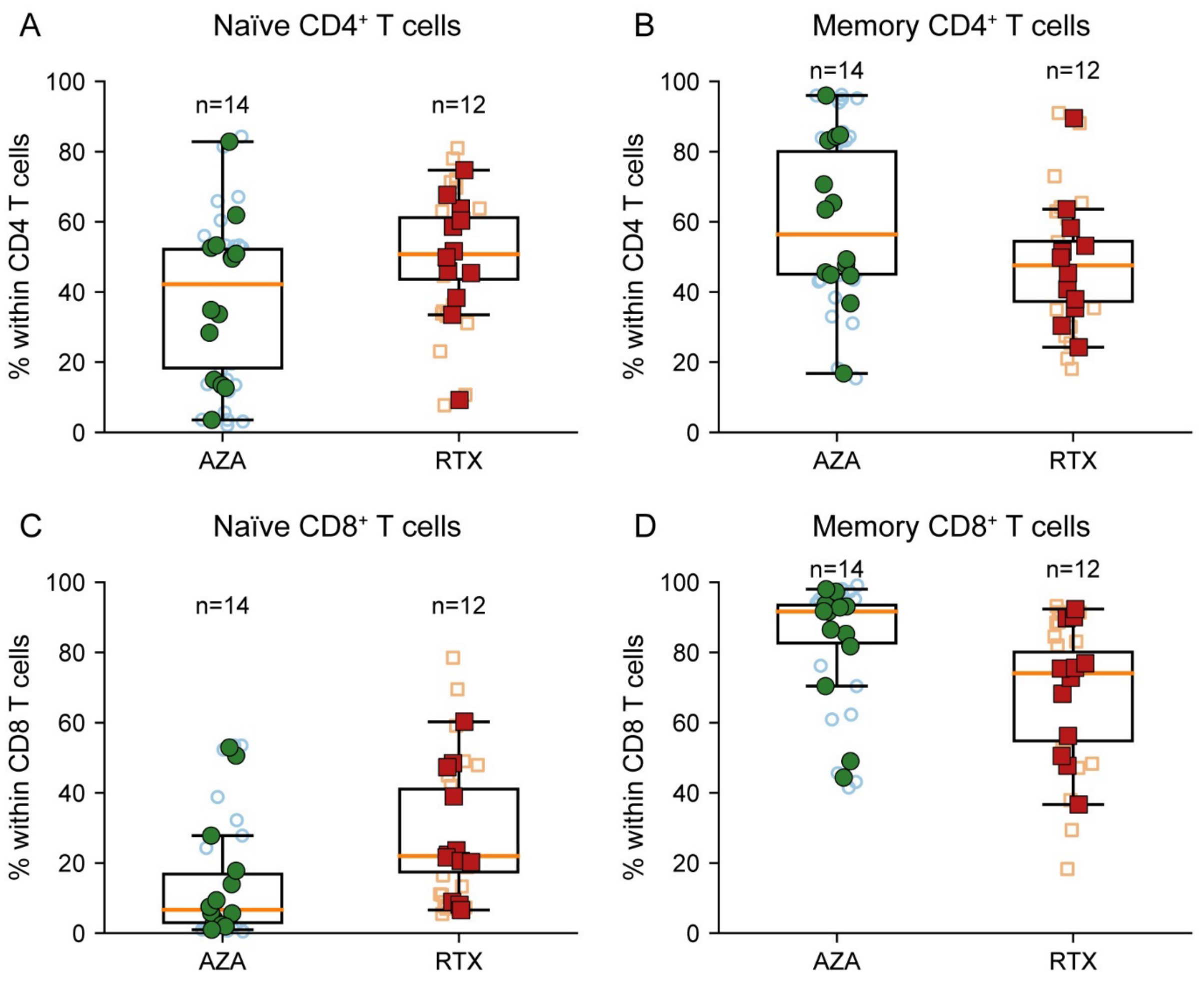

Figure S3. Conventional T-cell differentiation during remission

(A) Naive CD4$^+$ T cells expressed as % within CD4$^+$ T cells.

(B) Memory CD45RO+ CD4$^+$ T cells expressed as % within CD4$^+$ T cells.

(C) Naive CD8$^+$ T cells expressed as % within CD8$^+$ T cells.

(D) Memory CD45RO$^+$ CD8$^+$ T cells expressed as % within CD8$^+$ T cells.

Light symbols represent individual remission samples and filled symbols represent patient-level medians; boxplot elements are defined as in Figure 2. n indicates the number of patients contributing to each panel.

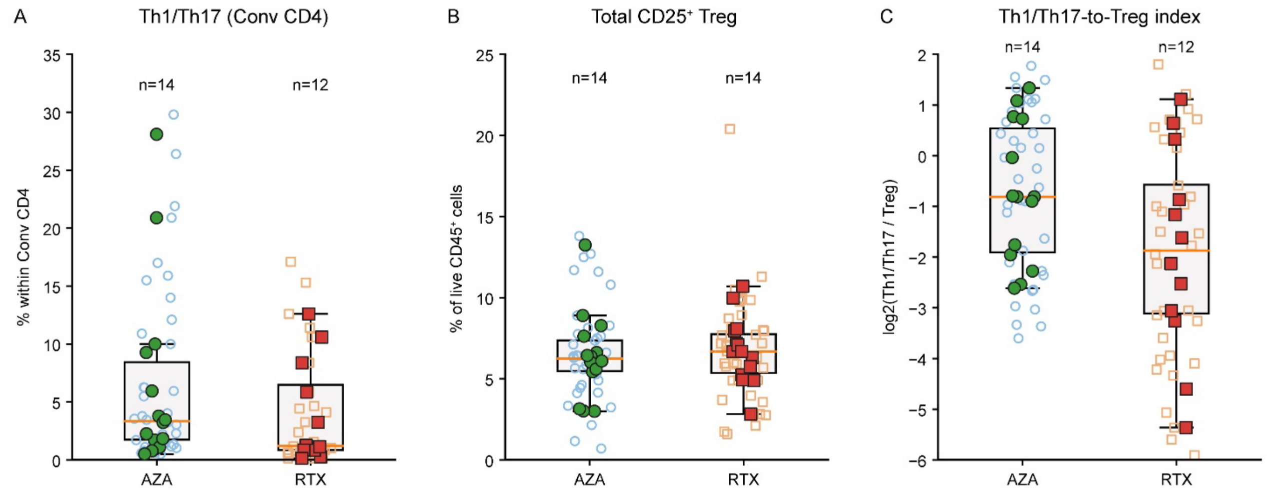

Figure S4. Effector-regulatory balance during remission

(A) Th1/Th17 ($CCR6^{+}CXCR3^{+}$) cells expressed as % within conventional $CD4^{+}$ T cells.

(B) Total $CD25^{+}$ Tregs expressed as % of live $CD45^{+}$ leukocytes.

(C) Composite balance index defined as log2[(Th1/Th17 [% of conventional CD4])/(total Treg [% of live $CD45^{+}$])].

Light symbols represent individual remission samples and filled symbols represent patient-level medians; boxplot elements are defined as in Figure 2. n indicates the number of patients contributing to each panel.

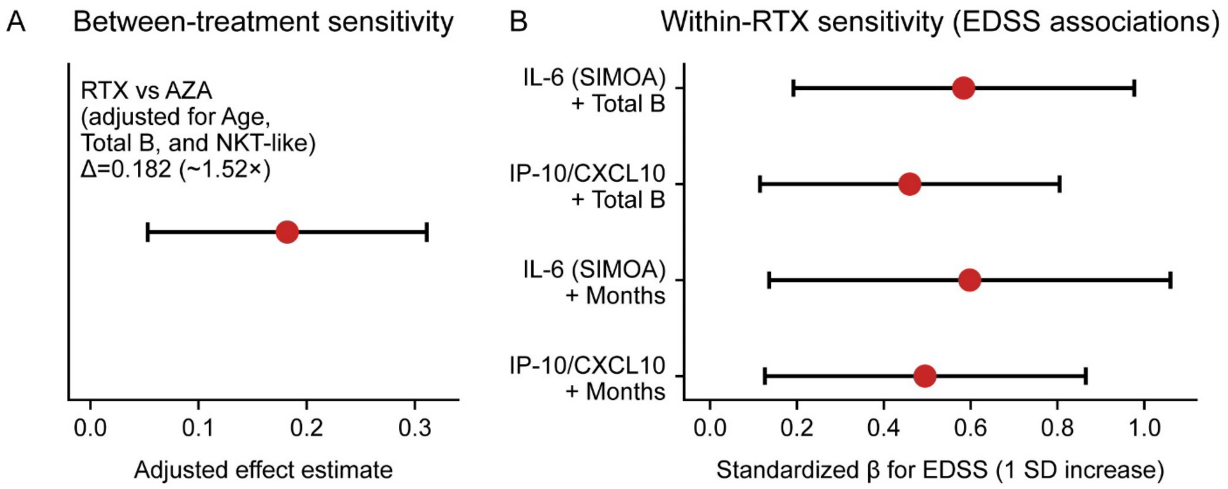

Figure S5. Additional robustness analyses

(A) Between-treatment sensitivity model for IP-10/CXCL10 comparing RTX versus AZA after adjustment for age, total B, and NKT-like cells.

(B) Within-RTX EDSS sensitivity models for IL-6 and IP-10/CXCL10 after additional adjustment for either total B burden or months after infusion.

Points indicate model estimates and horizontal lines indicate 95% confidence intervals. These additional robustness analyses are reported with nominal p values only, without FDR correction.
